# Microbiome “Inception”: an Intestinal Cestode Shapes a Hierarchy of Microbial Communities Nested within the Host

**DOI:** 10.1128/mbio.00679-22

**Published:** 2022-05-03

**Authors:** Jaelle C. Brealey, Laurène A. Lecaudey, Miyako Kodama, Jacob A. Rasmussen, Harald Sveier, Nolwenn M. Dheilly, Michael D. Martin, Morten T. Limborg

**Affiliations:** a Department of Natural History, NTNU University Museum, Norwegian University of Science and Technology (NTNU), Trondheim, Norway; b Center for Evolutionary Hologenomics, GLOBE institute, Faculty of Health and Medical Sciences, University of Copenhagen, Copenhagen, Denmark; c Laboratory of Genomics and Molecular Medicine, Department of Biology, University of Copenhagen, Copenhagen, Denmark; d Lerøy Seafood Group ASA, Bergen, Norway; e UMR 1161 Virology ANSES/INRAE/ENVA, ANSES Animal Health Laboratory, Maisons-Alfort, France; Cornell University

**Keywords:** Atlantic salmon, *Cestodes*, gut dysbiosis, holobionts, host-parasite-microbiome interactions, intestinal microbiomes

## Abstract

The concept of a holobiont, a host organism and its associated microbial communities, encapsulates the vital role the microbiome plays in the normal functioning of its host. Parasitic infections can disrupt this relationship, leading to dysbiosis. However, it is increasingly recognized that multicellular parasites are themselves holobionts. Intestinal parasites share space with the host gut microbiome, creating a system of nested microbiomes within the primary host. However, how the parasite, as a holobiont, interacts with the host holobiont remains unclear, as do the consequences of these interactions for host health. Here, we used 16S amplicon and shotgun metagenomics sequencing to characterize the microbiome of the intestinal cestode *Eubothrium* and its effect on the gut microbiome of its primary host, Atlantic salmon. Our results indicate that cestode infection is associated with salmon gut dysbiosis by acting as a selective force benefiting putative pathogens and potentially introducing novel bacterial species to the host. Our results suggest that parasitic cestodes may themselves be holobionts nested within the microbial community of their holobiont host, emphasizing the importance of also considering microbes associated with parasites when studying intestinal parasitic infections.

## INTRODUCTION

Microorganisms have been found to colonize the vast majority of multicellular life (1). It is now well established that the microbiome can modulate host phenotype (2, 3). The host immune system constantly interacts with the microbiome (4, 5), and there is mounting evidence that the underlying host genotype can influence microbiome composition (6, 7). Thus, emerging hologenomic theory posits that the genomes of the host and all its associated microorganisms are subject to coevolutionary forces and should be viewed as a “holobiont” rather than as independently evolving organisms (8). The dynamic interactions between a host and its microbiome have been most extensively studied in the gut, where the microbiome has been found to influence host adaptation (9–11), gene expression and regulation (12, 13), immune system maturation and modulation (14), maintenance of the gut mucosal barrier (15), and protection from pathogens (16, 17). Dysbiosis of the gut, defined as any change to the resident commensal gut microbiome relative to the community found in healthy individuals (18), is frequently associated with intestinal parasitic infections (19). However, it is not always clear whether dysbiosis increases susceptibility to parasite infection (20), the parasite infection triggers dysbiosis (21, 22), or a combination of both occurs (23).

There is growing recognition that multicellular parasites are themselves holobionts (24, 25). There are several well-documented cases of parasites hosting bacterial endosymbionts that are critical for parasite survival, like the dependence of filarial nematodes on their *Wolbachia* endosymbionts (26). Parasites thus represent an interesting case of one holobiont (the parasite) colonizing another holobiont (the parasite’s vertebrate host). This nested scenario is particularly true of intestinal parasites, which share physical space with the gut microbiome of the vertebrate host. In this case, the parasite-associated and host-associated microbiomes can be thought of as a continuum, transitioning from host-associated microbes residing in the host gut mucosa into host resident and transient microbes in the gut content, through to a combination of parasite and host microbes on or near the parasite body surface, and ending with internal parasite-specific microbes. While interactions between intestinal parasites, the host gut microbiome, and the host immune system have been characterized in previous studies (reviewed in Reynolds et al. [27]), few studies have taken into account how parasite-associated microbes might interact with the host and its associated microbes (24).

We exploited a unique, semicontrolled system to characterize microbial communities in an intestinal parasite, a cestode (or tapeworm) of the genus *Eubothrium*, and its definitive vertebrate host, Atlantic salmon (Salmo salar). Like most Platyhelminthes, members of *Eubothrium* have complex life cycles with intermediate hosts that are food sources of salmon (copepods and small pelagic fish), where the larvae develop in the body cavity, before reaching maturity in the salmon intestine after ingestion (28). Cestodes are responsible for significant health and economic burden in clinical, agriculture, and aquaculture settings (29–31). *Eubothrium* specifically impacts the salmon aquaculture industry, as intestinal infection of adult fish decreases growth and, in extreme cases, can be fatal (32, 33). We studied farmed adult salmon that were naturally infected with *Eubothrium* while housed in open seawater pens off the coast of southwestern Norway. This experimental setup allowed us to control many abiotic aspects that can affect salmon microbiome composition (e.g., feed, aquatic environment, genetic background) while still exposing the salmon to natural sources of microbes and pathogens. Cestodes lack a digestive tract and mouth, instead absorbing nutrients through their skin surface, the tegument; thus, microbes can only be associated with this tegument, or the internal body cavity. We therefore characterized the microbial communities sampled from the salmon-cestode interaction space, including the resident host microbiota of the salmon gut mucosa (“salmon gut”), the microbes loosely attached to the cestode tegument (disrupted with a phosphate-buffered saline [PBS] wash; “cestode wash”), and those associated with the cestode (whole body post-wash, including body cavity and tegument; “cestode body”). We also investigated dysbiosis in the salmon gut microbiome, comparing uninfected salmon to salmon with various levels of cestode infection. Finally, we shotgun sequenced, assembled, and functionally characterized the cestode body microbiome, generating genomes of two novel cestode-associated *Mycoplasma* bacteria. Our results suggest that parasitic cestodes should be considered holobionts with an associated microbiome when studying effects of intestinal parasitic infections.

## RESULTS AND DISCUSSION

### Cestode infection is associated with salmon gut dysbiosis.

**FIG 1:**
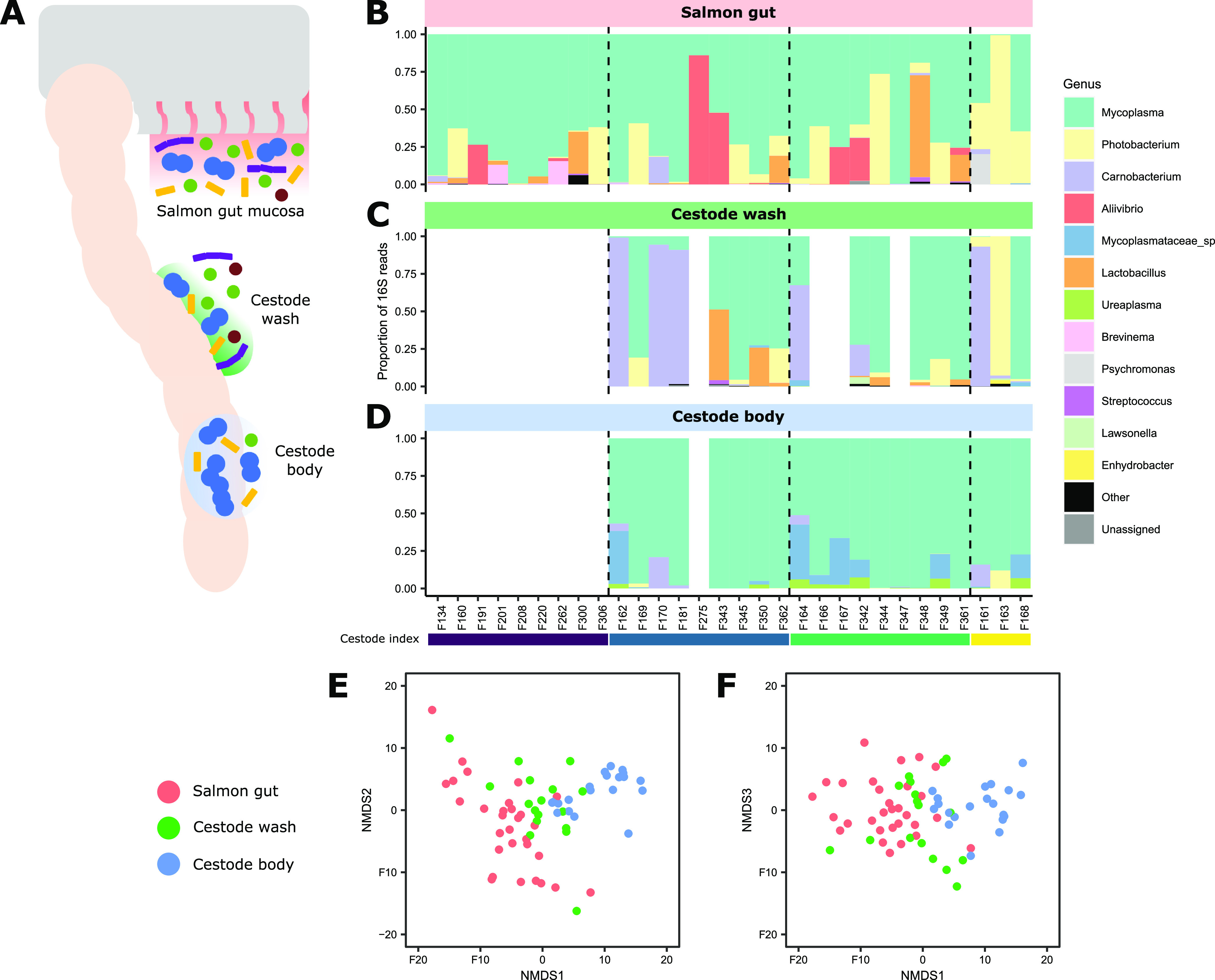
(A) Schematic of the three sampling sites, including a scrape of the salmon gut mucosa (red background), a PBS wash of the cestode tegument containing loosely attached microbes (green background), and the cestode body containing strongly attached or internal microbes (blue background). (B to D) Genus-level microbial composition of the salmon gut mucosa (B), cestode wash (C), and cestode body (D). The top 12 most abundant bacterial genera are colored, with the remainder grouped into “other.” ASVs that could not be assigned taxonomy at the genus level are grouped into “unassigned.” Individuals are ordered by cestode index (purple, 0; blue, 1; green, 2; yellow, 3), separated by the dashed vertical lines. (E and F) NMDS (*k* = 3) of Euclidean distances of ASV CLR-normalized abundances in samples from the salmon gut mucosa, cestode wash, and cestode body. Each point represents one sample from an individual, colored by sample type. NMDS stress, 0.117.

Cestode infection was observed in 378 (81.6%) of 463 adult harvest-ready salmon (Fig. S1 in the supplemental material). The degree of cestode infection was scored using an in-house semiquantitative veterinary index ranging from 0 (no observed cestode) to 3 (excessive [>3] numbers of cestodes impeding the passage of feed along the gastrointestinal tract) (Fig. S1). There was strong evidence that salmon size (gutted weight) decreased as the cestode index increased (Fig. S2), supporting results from earlier studies demonstrating negative growth effects in salmon associated with cestode infection (32, 33). Thirty salmon were selected for the cestode-related microbiome investigations, nine each with a cestode index of 0, 1, and 2 and three with a cestode index of 3. From these 30 salmon, we generated 1,172 amplicon sequence variants (ASVs) of the 16S rRNA gene (ASVs labeled seq1 to seq1172) from 30 host gut mucosal scrapings, 17 cestode wash samples, and 21 cestode body samples (Fig. 1A), of which 116 ASVs were retained after quality control.

10.1128/mbio.00679-22.3 FIG S1 Distribution of cestode indexes across fish housed in two sea pens, each fed a different commercial diet (*n* = 253 for feed 1 and *n* = 210 for feed 2). The semiquantitative cestode index is illustrated on the right, where 0 is no observed cestode, 1 is 1 to 3 visible cestodes, 2 is >3 cestodes but normal functioning of the gastrointestinal tract, and 3 is excessive numbers of cestodes that are likely impeding the passage of feed along the gastrointestinal tract. Download FIG S1, PDF file, 0.1 MB. Copyright © 2022 Brealey et al. 2022 Brealey et al. https://creativecommons.org/licenses/by/4.0/ This content is distributed under the terms of the Creative Commons Attribution 4.0 International license.

10.1128/mbio.00679-22.4 FIG S2 Weight of individuals decreases as cestode index increases. (a) Violin plot of gutted weight of salmon (kg) by cestode index in the entire cohort (*n* = 463). Horizontal lines represent the median. There was strong evidence that gutted weight decreased as cestode infection increased (*F*_5,456_ = 19.5, *P < *0.001, *R*^2^ = 0.17). (b) Box plot of gutted weight of salmon (kg) by cestode index in the 30 individuals selected for sequencing of cestode-related microbiomes in this study. There was some evidence that more heavily parasitized fish were smaller in this subset of individuals (*F*_5,24_ = 1.31, *P = *0.29, *R*^2^ = 0.051). Download FIG S2, PDF file, 0.05 MB. Copyright © 2022 Brealey et al. 2022 Brealey et al. https://creativecommons.org/licenses/by/4.0/ This content is distributed under the terms of the Creative Commons Attribution 4.0 International license.

Overall, the salmon gut mucosa was dominated by the genus *Mycoplasma* (Fig. 1B), a known commensal of the salmon intestinal microbiome (9, 34–38). However, there was evidence that the gut microbiota differed between uninfected and infected individuals. The cestode index accounted for 15.1% of the variation among individuals (Fig. S3), and there was weak evidence that alpha diversity in the gut mucosa increased as the cestode index increased (richness, *P = *0.11; Shannon, *P = *0.18) (Fig. S4). There was also weak evidence (false-discovery rate [FDR] < 0.2) that the relative abundance of *Mycoplasma* and the probable commensal *Brevinema* (37) decreased with increasing cestode infection, while the relative abundance of the putative pathobionts *Photobacterium* (39, 40), *Aliivibrio* (38, 40, 41), and *Carnobacterium* (42) increased (Fig. S5; Table S1). *Photobacterium* and *Aliivibrio* taxa in particular have been implicated in skin infections and septicemia of fish (38–41) and have been found at increased abundances in the guts of farmed Atlantic salmon during bacterial infections (38, 43). Our results therefore connect cestode infection to salmon gut dysbiosis, consistent with studies in other systems demonstrating altered host gut microbial communities during cestode infection (21, 44, 45).

10.1128/mbio.00679-22.1 TABLE S1 Associations between ASV or genus abundance and cestode index or sample type, as produced by MaAsLin2. Results with FDRs of <0.25 are included and those with FDRs of <0.05 are highlighted in gray. Download Table S1, PDF file, 0.2 MB. Copyright © 2022 Brealey et al. 2022 Brealey et al. https://creativecommons.org/licenses/by/4.0/ This content is distributed under the terms of the Creative Commons Attribution 4.0 International license.

10.1128/mbio.00679-22.5 FIG S3 NMDS (*k* = 2) of Euclidean distances of ASV CLR-normalized abundances in the salmon fish gut mucosa. Each point represents one individual, colored by cestode index. NMDS stress, 0.150. Cestode index accounted for 15.1% of the variation among individuals (PERMANOVA, F_3,26_ = 1.54, *P* = 0.035). Download FIG S3, PDF file, 0.01 MB. Copyright © 2022 Brealey et al. 2022 Brealey et al. https://creativecommons.org/licenses/by/4.0/ This content is distributed under the terms of the Creative Commons Attribution 4.0 International license.

10.1128/mbio.00679-22.6 FIG S4 Alpha diversity of each sample type from each individual, faceted by cestode index. (a) There was weak evidence that species richness (number of ASVs) increased as cestode index increased (*F*_4,25_ = 1.53, *P*_linear_ = 0.11, and *R*^2^ = 0.07 after controlling for sequencing effort). When considering all individuals irrespective of cestode index, there was strong evidence that alpha diversity decreased in the cestode body compared to the salmon gut mucosa (*F*_3,63_ = 6.04, *P = *0.0021, and *R*^2^ = 0.17 after controlling for sequencing effort). (b) There was also weak evidence that Shannon diversity (calculated using a Hill numbers framework) increased with cestode index (*F*_4,25_ = 0.93, *P*_linear_ = 0.18, *R*^2^ = −0.009 after controlling for sequencing effort) and decreased in the cestode body compared to the salmon gut (*F*_3,63_ = 1.49, *P = *0.061, and *R*^2^ = 0.022 after controlling for sequencing effort). (c) Venn diagram of ASVs detected in each sample type. The salmon gut microbiota has been separated into ASVs detected in nonparasitized salmon (cestode index = 0) and those in parasitized salmon (cestode index > 0). A total of 45 ASVs were unique to the salmon gut mucosa, irrespective of cestode detection; 31 were shared between salmon gut mucosa and cestode wash, 3 between salmon gut mucosa and cestode body, and 15 between all three sample types. Download FIG S4, PDF file, 0.3 MB. Copyright © 2022 Brealey et al. 2022 Brealey et al. https://creativecommons.org/licenses/by/4.0/ This content is distributed under the terms of the Creative Commons Attribution 4.0 International license.

10.1128/mbio.00679-22.7 FIG S5 Relative abundance (proportion of 16S reads) of taxa in the salmon gut mucosa that differed with cestode infection. (a) Genera that differed with cestode index. There was weak evidence that the relative abundance of *Mycoplasma* decreased with increasing cestode infection (effect size, −0.349 ± 0.118; FDR, 0.173) and the relative abundance of *Photobacterium* increased (effect size, 0.375 ± 0.112; FDR, 0.144). (b) Genera that differed by cestode presence. There was weak evidence that *Carnobacterium* and *Aliivibrio* had higher abundances in the gut mucosa of salmon infected with cestode (*Carnobacterium*, effect size, 0.018 ± 0.014; FDR, 0.506; and *Aliivibrio*, effect size, 0.078 ± 0.92; FDR, 0.779), and the likely commensal *Brevinema* had higher abundance in the gut mucosa of uninfected salmon (effect size, −0.041 ± 0.014; FDR, 0.248). (c) Relative abundance of ASVs that differed with cestode index (FDR, <0.25; statistics provided in Table S1). Note that relative abundance is shown on different scales within each plot in panel c. Download FIG S5, PDF file, 0.02 MB. Copyright © 2022 Brealey et al. 2022 Brealey et al. https://creativecommons.org/licenses/by/4.0/ This content is distributed under the terms of the Creative Commons Attribution 4.0 International license.

### Nested microbial communities within the host holobiont.

The salmon gut mucosa and cestode body microbiota were clearly differentiated, with the microbiota associated with the cestode wash being intermediate (Fig. 1E and F). Sample type explained 21.4% of the variation among samples (permutational multivariate analysis of variance [PERMANOVA], *F*_2,64_ = 8.71, *P = *0.001), although the variation within the cestode body was less than within the other two sample types (*F*_2,64_ = 6.57, *P = *0.005). Alpha diversity decreased in the cestode body compared to the salmon gut mucosa after controlling for sequencing effort (richness, *P = *0.0021; Shannon, *P = *0.061) (Fig. S4). Twenty-two ASVs were only detected in cestode-derived samples (wash or body) (Fig. S4). The salmon gut mucosa included 45 unique ASVs (10 of which were only detected in uninfected fish), 31 ASVs also present in the cestode wash, and 18 ASVs shared with the cestode body or all three sample types (Fig. S4). The mycoplasmas (*Mycoplasma*, *Ureaplasma*, and uncharacterized *Mycoplasmataceae* species) dominated the cestode body, while the cestode wash predominantly included *Mycoplasma*, *Carnobacterium*, or *Photobacterium* (Fig. 1B; Fig. S6; Table S1). Twenty-three ASVs had increased abundance in one of the three sample types, particularly mycoplasma ASVs in the cestode body, *Mycoplasma* and *Carnobacterium* ASVs in the cestode wash, and ASVs related to *Photobacterium* and *Brevinema* in the fish gut mucosa (Fig. S6; Table S1).

10.1128/mbio.00679-22.8 FIG S6 Relative abundance (proportion of 16S reads) of genera (a) and ASVs (b) that differed with a particular sample type (FDR < 0.2; statistics provided in Table S1). ASVs are ordered by genus (alphabetically). Note that relative abundance is shown on different scales within each plot in panel b. Download FIG S6, PDF file, 0.1 MB. Copyright © 2022 Brealey et al. 2022 Brealey et al. https://creativecommons.org/licenses/by/4.0/ This content is distributed under the terms of the Creative Commons Attribution 4.0 International license.

**FIG 2:**
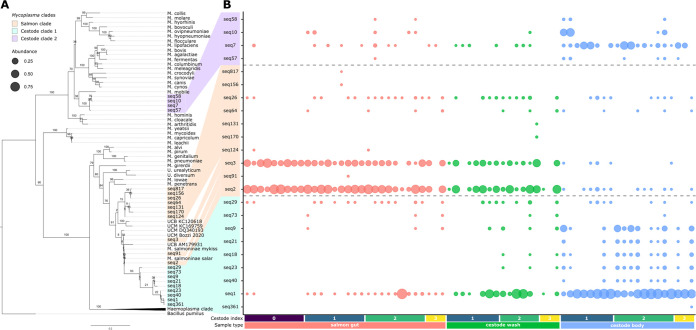
(A) Phylogeny of *Mycoplasma*-related ASVs (*Mycoplasma*, *Ureaplasma*, and uncharacterized *Mycoplasmataceae* species, labeled with “seq” prefix) with reference *Mycoplasma* species. The maximum-likelihood tree was constructed using RAxML with rapid bootstrapping (1,000 bootstraps). The tree was rooted using the outgroup Bacillus pumilus. The nodes are labeled with the bootstrap values. The *Haemoplasma* clade of *Mycoplasma* has been collapsed and truncated for visualization. (B) Presence and relative abundance of *Mycoplasma*-related ASVs in each sample. Samples are colored by sample type. Within each sample type, samples are ordered by cestode index. Circle area scales with the relative abundance of each ASV. The labels of ASVs in each of the three *Mycoplasma* clades are highlighted.

**FIG 3:**
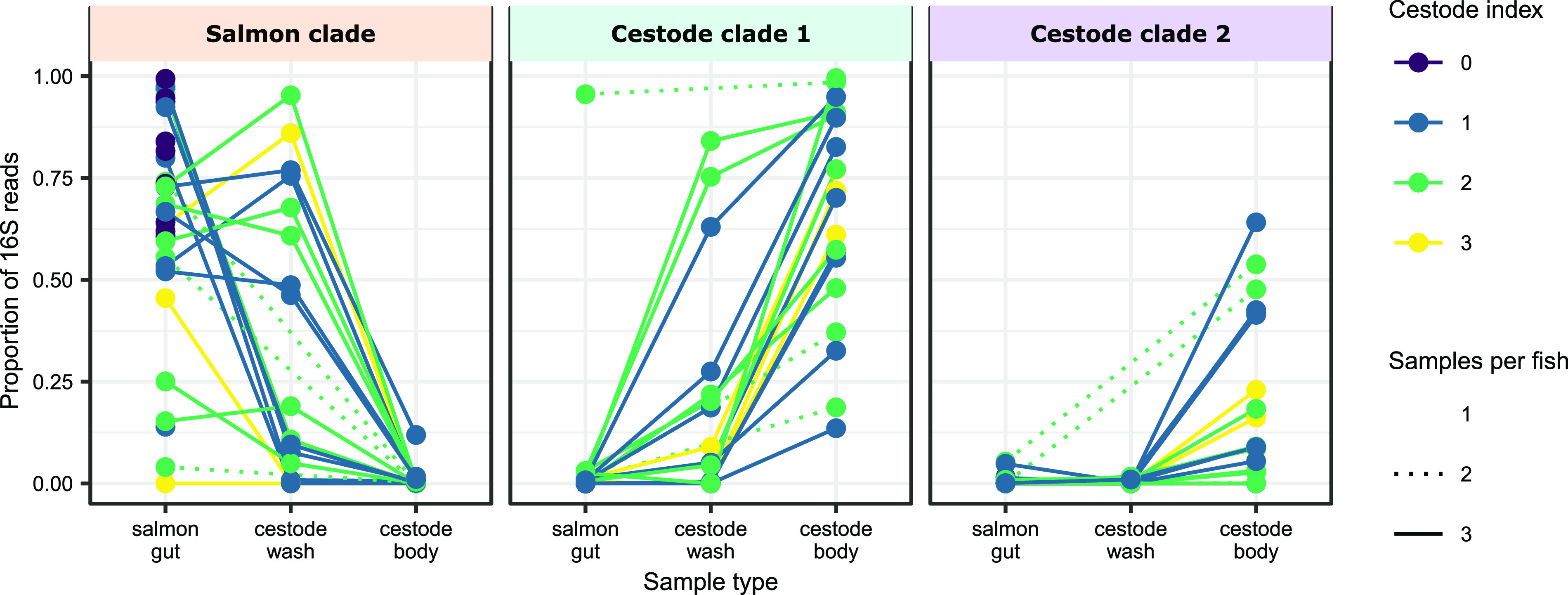
Change in relative abundance of the three *Mycoplasma* clades (as indicated on the phylogenetic tree in Fig. 2) between each sample type. Solid lines connect each of the 3 samples taken from each individual. Dashed lines indicate individuals where one of the two cestode samples was missing. Individuals are colored by cestode index.

We identified one set of mycoplasma ASVs that were associated with the salmon gut mucosa and another set that were associated with the cestode body (Fig. 2; Table S1). The salmon-associated mycoplasma ASVs were closely related to the *Mycoplasma* 16S sequence(s) described in previous studies of the gut microbiota in Atlantic salmon (9, 38, 46) and other marine fish (47–49) (Fig. 2A). The cestode-associated mycoplasma ASVs clustered into two clades, one sister clade to salmon-associated mycoplasma and another, more divergent clade clustering close to the known fish pathogen Mycoplasma mobile (22, 50, 51) (Fig. 2A, cestode clades 1 and 2, respectively). Within an individual, the salmon mycoplasma clade was frequently at the highest abundance in the gut mucosa and at the lowest abundance in the cestode body, while the opposite pattern was observed for the two cestode-associated clades (Fig. 3).

Separation of host- and cestode-specific microbes is difficult due to the possibility of contamination of cestode samples by host microbiota during sampling (52). We rinsed gut content from both the salmon mucosa and the cestode before sampling. The cestode tegument was sampled by vigorous shaking in PBS, forming the cestode wash samples and likely containing a mixture of loosely attached resident and transient microbes from the salmon gut content, as well as potentially cestode-associated microbes, as suggested by the “intermediate” state of the cestode wash microbiota composition compared to the salmon mucosa and cestode body microbiota. The washed cestode body was then sampled to identify microbes either strongly attached to the surface or internal to the cestode. In future studies, a serial washing procedure could be used to sample the cestode tegument and body cavity microbiota to improve the likelihood of identifying cestode-specific microbes after the final wash (52, 53).

Our results suggest that cestodes harbor specific microbes that are not at high abundances in the host gut mucosa, although with our sampling procedure, we cannot determine whether they are external (i.e., on the tegument) or internal (i.e., inside the body cavity). These findings are consistent with studies of other cestodes and related helminths that parasitize fish, which show that a unique cestode microbiota can be observed at each life stage, including during colonization of the body cavity of intermediate hosts (21, 54). However, as adults in the intestine of the definitive host, the microbiota of these helminths tend to be more similar to the microbiota of the host gut environment (52, 54). Our study demonstrated that the microbial communities associated with adult cestodes in our system are dominated by bacteria that are of the same family (the mycoplasmas), but of distinct clades, as those residing in the host gut mucosa. These results suggest that while the three mycoplasma clades can be detected in any of the three sample types investigated, divergent selection seems to occur at the cestode and the salmon mucosa for specific mycoplasma phylotypes, reflecting a nested system of microbial communities.

### Identification of different *Mycoplasma* phylotypes in host and parasite.

To further investigate this mycoplasma strain variation, we generated metagenomes from eight cestode body samples and *de novo* assembled three *Mycoplasma* metagenome-assembled genomes (MAGs) (Table S2). Each of these MAGs included a 16S sequence that exactly matched one of the three *Mycoplasma* ASVs dominating the cestode body amplicon sequencing data set (i.e., seq1 from cestode clade 1, here referred to as “CE_seq1,” and seq7 and seq10 from cestode clade 2, here “CE_seq7” and “CE_seq10,” respectively). Based on the presence of universal marker genes, the CE_seq1 and CE_seq7 MAGs were estimated to be 77.7% and 78.0% complete, respectively, whereas CE_seq10 was 38.0% complete (Table S2). We compared these novel cestode-related MAGs to *Mycoplasma* MAGs previously recovered from salmonids (9) (“*Candidatus* Mycoplasma salmoninae salar” from Atlantic salmon, “*Candidatus* Mycoplasma salmoninae mykiss” from rainbow trout, and “*Candidatus* Mycoplasma lavaretus” from European whitefish), as well as five closely related *Mycoplasma* and *Ureaplasma* references (M. iowae, M. mobile, M. penetrans, U. diversum, and U. urealyticum).

10.1128/mbio.00679-22.2 TABLE S2 Statistics and ENA accessions of the three *Mycoplasma* MAGs generated from the cestode body shotgun metagenome. Download Table S2, PDF file, 0.1 MB. Copyright © 2022 Brealey et al. 2022 Brealey et al. https://creativecommons.org/licenses/by/4.0/ This content is distributed under the terms of the Creative Commons Attribution 4.0 International license.

**FIG 4:**
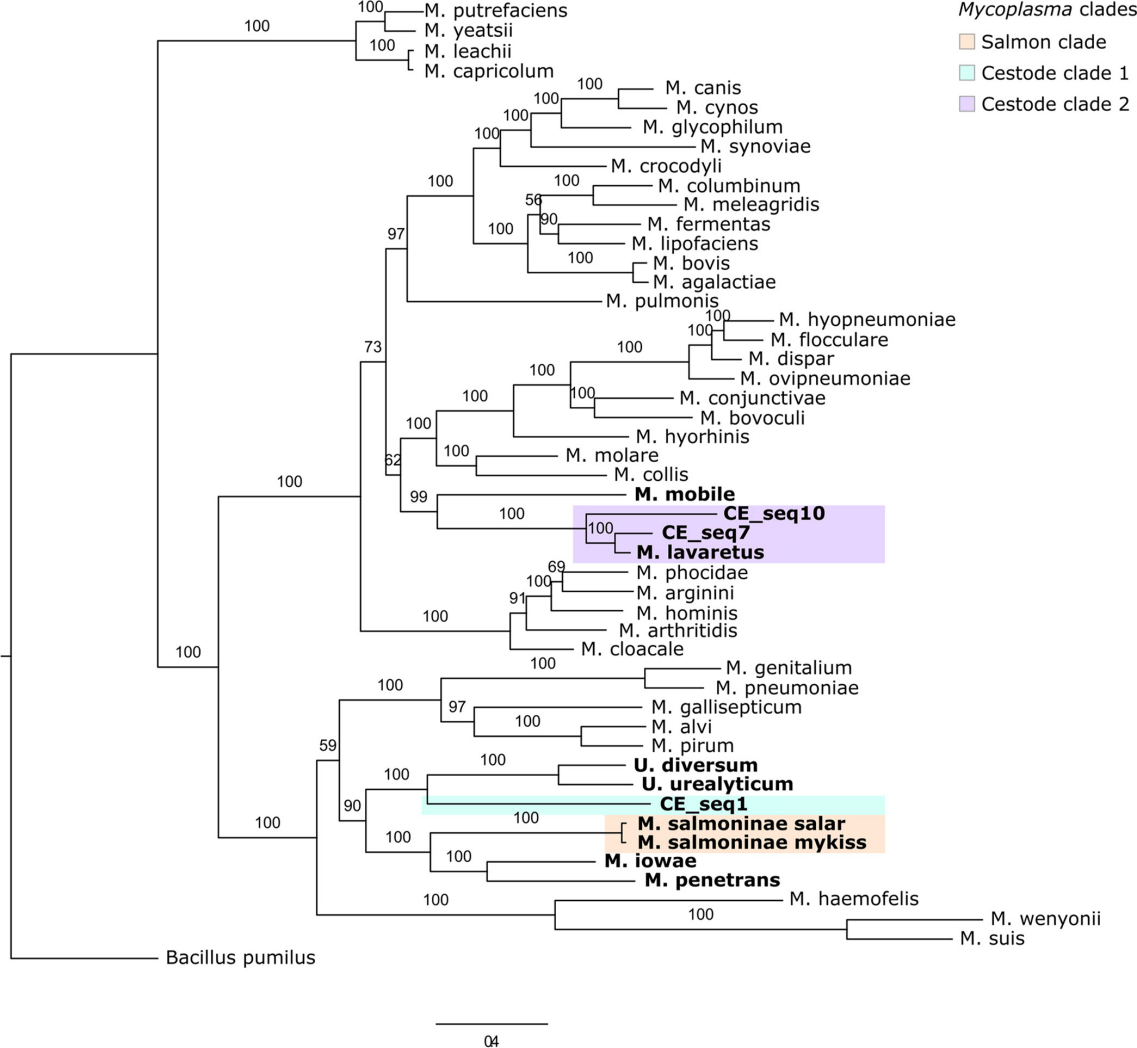
Phylogenomic tree of cestode *Mycoplasma* MAGs (CE_seq1, CE_seq7, and CE_seq10) and salmonid “*Ca.* Mycoplasma salmoninae” and “*Ca.* Mycoplasma lavaretus” MAGs (9) with reference *Mycoplasma* and *Ureaplasma* genomes. The maximum-likelihood tree was constructed based on concatenated single-copy core bacterial genes using RAxML with rapid bootstrapping (100 bootstraps). The tree was rooted using the outgroup Bacillus pumilus. The nodes are labeled with the bootstrap values. Genomes included in the functional comparison are in bold. As in Fig. 2, the salmonid *Mycoplasma* clade is highlighted in orange, cestode-associated clade 1 in cyan, and cestode-associated clade 2 in light purple.

Phylogenomics using concatenated bacterial single-copy genes present in all genomes generally recapitulated the relationships observed in the 16S tree (Fig. 4). CE_seq1 was related to, but distinct from, the salmon-associated “*Ca.* Mycoplasma salmoninae” MAGs (70.8% average nucleotide identity [ANI] compared to “*Ca.* Mycoplasma salmoninae salar” and 70.9% to “*Ca.* Mycoplasma salmoninae mykiss”) and their relatives M. penetrans and M. iowae. Instead, CE_seq1 clustered with the *Ureaplasma* references. CE_seq7 and CE_seq10 were closely related to “*Ca.* Mycoplasma lavaretus” (CE_seq7, 81.0% ANI; CE_seq10, 83.9% ANI), with some similarity to the fish pathogen M. mobile. Based on these results, we suggest that CE_seq1 is a new species of *Mycoplasma*, while CE_seq7 and CE_seq10 may be subspecies of “*Ca.* Mycoplasma lavaretus.”

The presence of these novel *Mycoplasma* species at high abundance in the cestode body microbiota and low abundance in the salmon gut mucosa supports the 16S results (Fig. 3), suggesting that divergent selection is occurring at the cestode and the salmon mucosa for specific *Mycoplasma*. The phylogenetic position of the cestode MAGs relative to “*Ca.* Mycoplasma salmoninae” demonstrates the establishment of distinct, yet closely related *Mycoplasma* phylotypes within their respective hosts, suggesting a specific interaction between salmonid hosts, cestodes, and *Mycoplasma* species and, thus, potential for coevolutionary relationships.

### Novel cestode *Mycoplasma* genomes suggest holobiont-specific adaptations.

To gain insights into the adaptation of the cestode *Mycoplasma* to their environment, we functionally characterized the two nearly complete cestode-associated *Mycoplasma* MAGs and compared them to the three salmonid-associated *Mycoplasma* MAGs. The mycoplasmas have highly specific adaptations for survival in their respective hosts (55). The cestode has limited metabolic capacity and relies on the host for obtaining nutrients (56); thus, microbial survival inside the cestode body or on the cestode tegument may require specialized adaptations compared to microbes adapted to the salmon gut. Most gene clusters in CE_seq1 were unique (586 of 741, 79.1%), while only 152 (20.5%) were shared with at least one of the salmonid-associated MAGs (Fig. S7), suggesting that CE_seq1 may be more adapted for colonization of cestodes than survival in the salmon gut. In contrast, fewer gene clusters were unique to CE_seq7 (192 of 642, 29.9%); instead, 447 (69.6%) were shared with at least one of the salmonid-associated MAGs, mostly “*Ca.* Mycoplasma lavaretus” (Fig. S7).

10.1128/mbio.00679-22.9 FIG S7 Functional annotation of cestode-associated *Mycoplasma* MAGs. (a) Venn diagram of gene clusters detected in cestode-associated and salmonid-associated *Mycoplasma* MAGs. Gene cluster counts unique to CE_seq1 are highlighted in cyan, unique to “*Ca.* Mycoplasma salmoninae” (“*Ca.* Mycoplasma salmoninae salar” and/or “*Ca.* Mycoplasma salmoninae mykiss”) in orange, and those unique or shared between CE_seq7 and “*Ca.* Mycoplasma lavaretus” in shades of lilac. Consistent with phylogenetic patterns observed in Fig. 4, CE_seq7 and “*Ca.* Mycoplasma lavaretus” share many gene clusters (361), while CE_seq1 has few gene clusters in common with the “*Ca.* Mycoplasma salmoninae” MAGs (3 + 71 + 3 = 77), instead containing the largest number of unique gene clusters (586). (b) Probable energy production pathways detected in cestode (CE_seq1 and CE_seq7) and three previously described salmonid (“*Ca.* Mycoplasma salmoninae salar,” “*Ca.* Mycoplasma salmoninae mykiss,” and “*Ca.* Mycoplasma lavaretus”) *Mycoplasma* MAGs. Glycolysis via the EMP pathway, glycerol metabolism, and the arginine deiminase pathway of arginine degradation is shown. The major steps generating important by-products (ATP or NH_3_) are shown with dashed gray arrows. For each gene in the pathway, colored boxes indicate that the gene was successfully annotated in the respective MAG (white boxes indicate that the gene was not detected in the respective MAG). Dotted lines represent unknown steps in the pathway. Download FIG S7, PDF file, 0.6 MB. Copyright © 2022 Brealey et al. 2022 Brealey et al. https://creativecommons.org/licenses/by/4.0/ This content is distributed under the terms of the Creative Commons Attribution 4.0 International license.

Following functional annotation of these gene clusters, we identified differences in key metabolic pathways (Fig. S7). While the three salmonid MAGs and CE_seq7 had a complete glycolysis pathway, which generates ATP through carbohydrate degradation, CE_seq1 lacked many of the core components of glycolysis (although we cannot exclude the possibility that we do not observe these components due to the genome incompleteness [77.7%] of CE_seq1). However, CE_seq1 and the salmonid MAGs had a complete arginine deiminase pathway, which was missing in CE_seq7. This reversible pathway can either use ATP and ammonium to synthesize arginine or produce energy through arginine degradation, and it likely reflects an adaptive trait of salmonid *Mycoplasma* species to survive in the ammonia-rich salmon gut (9).

CE_seq7 and “*Ca.* Mycoplasma lavaretus” also had an incomplete glycerol metabolism pathway (Fig. S7), including the presence of the glycerol uptake facilitator protein GlpF, suggesting an ability to acquire environmental glycerol to feed into glycolysis, among other pathways. Glycerol metabolism has been highlighted as an important virulence factor of pathogenic *Mycoplasma* species, as the oxidation of the downstream product glycerol-3-phosphate by an oxidase (GlpO) can generate reactive oxygen species like hydrogen peroxide that may be detrimental to the host (57). However, the same reaction can also be catalyzed by a dehydrogenase (e.g., gpsA) without generating hydrogen peroxide by-products. In all cestode- and salmonid-associated *Mycoplasma* MAGs, only the dehydrogenase was identified (Fig. S7); thus, it is unclear whether glycerol metabolism would promote virulence in these strains.

In the phylogenetic analysis, we observed that CE_seq7 shares some similarity to M. mobile, a known fish pathogen infecting the host via the gills (50). Combined with the possibility of glycerol metabolism as a virulence factor, these results raise the question of whether cestode-associated microorganisms like CE_seq7 could contribute to disease states in the salmon. There is evidence in other helminths and protozoan parasites that such obligate parasites can transfer bacterial endosymbionts to their hosts, where the bacteria can cause disease (58–60). Thus, the infectious cestode may act as a vector, introducing microbial species into the salmon gut, which can then interact with the host holobiont and affect host health. Furthermore, the vector of the cestode itself (i.e., the intermediate copepod host), may also play a role in introducing novel microbes to the salmon gut, highlighting the importance of future studies characterizing the host- and parasite-associated microbiomes at each stage of the parasite life cycle (24).

Overall, our functional comparison was limited due to the lack of functional studies of *Mycoplasma* that colonize nonmammalian hosts (61); thus, we were unable to further predict whether the cestode-associated *Mycoplasma* MAGs contain genes that aid colonization and/or pathogenesis in either the cestode or the salmon gut. It is also currently unclear whether these MAGs are symbionts with functional roles in the cestode. Thus, further studies focusing on functional interactions between parasite and microbe are needed to determine whether these microbes have a phenotypic impact on the cestode host.

### Conclusions.

Parasitic helminths, like cestodes, are highly successful parasites, infecting most vertebrate taxa, including humans. They are responsible for significant health and economic burden in clinical, agriculture, and aquaculture settings (29–31). Pharmaceutical treatments are currently limited, and parasite resistance to antiparasitic drugs is increasing (29). It is therefore of paramount importance to understand host-helminth interactions that explain host susceptibility and resistance to better predict and treat parasite infections. In this study, we demonstrate that an intestinal cestode harbors its own external and/or internal microbiome. Future studies should determine the biological roles of these microbes in cestode survival and reproduction. We also establish that parasite infection is associated with host gut dysbiosis through the introduction of, and/or selection for, putatively detrimental microbes. Our study illustrates that when studying intestinal parasite infections, the gut microbial environment should be considered to have a nested, hierarchical structure, including both host-associated and parasite-associated microbes that may interact, with positive or negative consequences for the host. Beyond broad biomedical importance for both human and animal health, such an approach also has interesting implications for understanding parasite ecology and evolution (24).

## MATERIALS AND METHODS

### Sampling.

Samples used for this study were obtained as part of the HoloFish project (Norwegian Seafood Research Fund; project no. 901436). We sampled 463 ready-to-harvest Atlantic salmon from a commercial production site close to Bergen, Norway (60°29′58.9″N, 4°55′41.3″E) owned by Lerøy Seafood Group during April 2018. All salmon were sampled in accordance with Norwegian regulations (FOR-2015-06-18-761, The Norwegian Ministry of Agriculture and Food) (details in Text S1 in the supplemental material). All salmon were from the same commercial broodstock and production cohort and were thus reared under identical environmental conditions throughout their life cycle. Samples were obtained from two groups reared in separate sea pens and fed two different standard commercial diets (feed 1 and feed 2). These diets have been anonymized but were provided by BioMar and EWOS (produced in 2018). Inclusion of two commercial diets allowed us to replicate observations on the effect of cestode infections while controlling for specific feed effects on the infection status. Gutted weight (kg) for each fish was recorded to assess the association between overall lifetime growth of each fish and cestode infection status at the time of sampling. We also recorded the intestinal cestode infection status for each of the 463 salmon using a cestode index from 0 to 3 where 0 represents no observed cestode, 1 signifies 1 to 3 visible cestodes, 2 represents >3 cestodes but normal functioning of the gastrointestinal tract, and 3 represents excessive numbers of cestodes that are likely impeding the passage of feed along the gastrointestinal tract (Fig. S1). This index, developed by experienced Lerøy veterinarians and commonly used as part of fish health assessments in aquaculture farms (C. Kalgraff and H. Sveier, personal communication), provides a semiquantitative assessment of infection with functional consequences for fish health.

We characterized three microbial communities from the salmon intestine (salmon gut mucosa, cestode wash, and cestode body). We sampled 21 salmon infected with cestodes (cestode index from 1 to 3), as well as 9 uninfected salmon (cestode index of 0), with an equal representation of each commercial feed type. For the salmon gut sample, gut content was removed, and the intestine washed in a saline water solution before scrapes of the gut mucosal layer were taken and stored in 1× DNA/RNA Shield buffer (Zymo Research). For each infected salmon, one cestode was first rinsed in saline to remove visible gut content contamination and then placed into a phosphate-buffered saline (PBS) solution and hand shaken for at least 20 s to collect microbes loosely attached to the tegument. The cestode was then preserved separately in 96% EtOH for analysis of the cestode body microbiome. All samples were stored at −20°C until DNA extraction.

### DNA extraction.

We extracted DNA from 30 salmon gut mucosa samples. For the 21 parasitized salmon, we also extracted DNA from their associated cestode wash and cestode body samples. In parallel, we extracted four mock community samples as positive controls, using ZymoBIOMICS microbial community standard (Zymo Research) as an input. This mock community comprises eight bacterial strains and two fungal strains that are not expected to be part of the salmon gut microbiome. We also extracted four extraction blanks (sterile water or extraction buffer) as negative controls. The positive and negative controls were all processed in the same manner as the rest of the samples. All extractions were performed using the DNeasy PowerLyzer PowerSoil kit (Qiagen) following the manufacturer’s protocol. Before PCR amplification, we treated each sample with the OneStep PCR inhibitor removal kit (Zymo Research).

### 16S library build and sequencing.

To amplify the V3-V4 region of the 16S rRNA gene, we used bacterium-specific custom primers, modified from the standard 341F and 806R primers (62), for a two-step PCR-based approach with Illumina Nextera dual indexes (Text S1). Briefly, PCR amplification with 35 or 40 cycles was performed in duplicate to control for putative bias resulting from random PCR noise. The resulting PCR products were purified using solid-phase reversible immobilization (SPRI) bead purification (63). To incorporate the Nextera dual indexes, we performed a second-stage PCR of eight cycles with a unique index combination for each sample and replicate. PCR products were purified using SPRI beads, and the DNA concentration was measured with a Qubit 2.0 fluorometer (Thermo Fisher Scientific). Libraries were pooled in equimolar ratios, including positive- and negative-control libraries for downstream quality control and contaminant filtering. Sequencing was performed at the Danish National High-throughput Sequencing Centre (University of Copenhagen, Denmark) on the Illumina MiSeq (reagent kit v3) to generate 300-bp paired-end reads. To mitigate the effects of sequencing low‐complexity libraries and to improve the clustering, 10% PhiX was added to pools prior to sequencing.

### Metagenomics library build and sequencing.

Eight cestode body samples were selected for shotgun metagenomic sequencing of the cestode-associated microbiome. Metagenomic library preparation and sequencing were carried out as previously described for salmonid-related *Mycoplasma* (9). Briefly, fragmentation of DNA was carried out in Covaris microTube-50 AFA fiber screw-cap tubes using a Covaris M220 focused ultrasonicator. DNA mass for each sample was normalized to 200 ng prior to library preparation. Library preparation was carried out following the blunt-end single-tube method of library preparation for degraded DNA (64, 65). Prior to indexing of libraries, all libraries were analyzed with quantitative PCR (qPCR) to estimate optimal cycle settings on an Mx3005P qPCR system (Agilent Technologies). Indexed libraries were quality controlled with a Bioanalyzer 2100 (Agilent Technologies) and sequenced on an MGISEQ-2000RS at BGI Europe (Copenhagen, Denmark), generating 150-bp paired-end reads.

### Data processing and taxonomy assignment for 16S profiling.

Adapter sequences were trimmed from 3′ ends of demultiplexed forward and reverse reads using AdapterRemoval v2.3.1 (66) with default parameters. The DADA2 v1.17.3 (67) package was used for the remainder of the processing steps with R v4.0.2, including quality filtering and trimming, dereplication, inference of ASVs, and chimera filtering (Text S1). Taxonomy was then assigned at the genus level using a custom database based on the SILVA nonredundant SSU v138 training set provided by DADA2 (Text S1). Species assignments at 100% sequence identity were performed where possible.

Further processing was conducted in RStudio v1.3.959 using phyloseq v1.32.0 (68). To remove PCR false positives, we only retained ASVs detected at least twice in both PCR replicates. Putative contaminant sequences were identified and removed with decontam v1.8.0 (69) using both the frequency and prevalence functions. Several ASVs detected in the blanks originated from the mock control samples, indicating low-level cross-contamination had occurred between samples (70). We therefore filtered all ASVs present in the mock samples that matched the known (genus-level) composition of the mock community. Finally, we removed all eukaryotic, chloroplast, and mitochondrial ASVs. After these processing steps, salmon and cestode sample replicates retained, on average, 39,355 sequences (lowest sequencing effort, 1,014 sequences; highest sequencing effort, 99,151 sequences). Species accumulation curves indicated that sequencing reached saturation for most samples (see additional figures at https://github.com/jcbrealey/cestode_microbiome). The mock samples had a genus-level composition that matched the theoretical bacterial community composition (see additional figures on GitHub). There was good concordance between PCR replicates of both the mock and the salmon and cestode samples (see additional figures on GitHub). We therefore merged the ASV count data for each replicate in a sample using the phyloseq function merge_samples.

### Statistical analysis.

For all analyses, cestode index was treated as an ordered factor, while sample type (salmon gut, cestode wash, or cestode body) was treated as an unordered factor. Associations between cestode index and gutted fish weight were evaluated in linear regression, including feed type and sex as covariables. For visualization of 16S data, relative abundance was calculated at the ASV level and summed at the genus level. To account for the compositional nature of microbiome sequence data (71), for statistical analyses, ASV and genus counts were transformed with a pseudocount of 1 and the centered log ratio (CLR) calculated using the R package microbiome (http://microbiome.github.io/microbiome). Nonmetric multidimensional scaling (NMDS) was performed using CLR-normalized abundance values and Euclidean distance with the ordinate function in phyloseq. The NMDS stress value, which is a measure of the degree to which the distance between samples in the reduced dimensional space corresponds with the actual distance between samples (similar to a goodness-of-fit value), has been included in the figure legend of each NMDS plot. PERMANOVAs were performed on the Euclidean distances using the adonis function in the R package vegan (https://github.com/vegandevs/vegan). Homogeneity of group dispersions was assessed using the betadisper and permutest functions. Species richness was calculated using the specnumber function in vegan and compared among groups using linear regression. Shannon diversity was calculated using a Hill numbers framework with the R package hillR (*q *= 1) (72). MaAsLin2 (73) was used to identify changes in ASV- and genus-level relative abundances associated with sample type (fixed effect, salmon gut as reference level), with individual ID and sampling date included as random effects. To identify changes in relative abundance associated with cestode index, MaAsLin2 was run with cestode index, feed type, sex, and size category (small category as reference level) as fixed effects and sampling date as a random effect. The same model was also run using cestode presence as a binary variable (yes/no) instead of cestode index, but no taxon was associated with feed type, sex, or size at false-discovery rate (FDR) of <0.2. When interpreting statistical tests, we have used “language of evidence” as outlined in Muff et al. (74). Thus, *P* values and FDRs of <0.05 are interpreted as providing moderate or strong evidence for the respective association, while values between 0.05 to 0.2 are interpreted as providing little or weak evidence.

### Phylogenetic tree based on 16S profiling.

ASVs classified as *Mycoplasmataceae* (unknown genus), *Mycoplasma*, or *Ureaplasma* were placed in a phylogenetic tree with a reference set of mycoplasma 16S sequences (*Mycoplasma*, *Ureaplasma*) and Bacillus pumilus as an outgroup to root the tree (accessions available at https://github.com/jcbrealey/cestode_microbiome). Using QIIME v2-2020.8 (75), sequences were aligned with MAFFT (76), and uninformative positions were masked (--p-max-gap-frequency 0.2, --p-min-conservation 0.4). A bootstrapped, maximum-likelihood phylogenetic tree was constructed with RAxML rapid bootstrapping (77, 78), using the GTRGAMMA substitution model and 1,000 bootstrap replicates. The resulting tree was visualized in FigTree v1.4.3 (https://github.com/rambaut/figtree/).

### Metagenomic data processing and MAG generation.

Processing steps of the shotgun metagenomics data included adapter removal (66), PCR duplicate removal (79), and filtering of reads mapping to the sequencing control phiX174, human, salmon (80), and cestode reference genomes (81–84) (Text S1). A coassembly of reads from all samples was then performed with MEGAHIT v1.1.1 (85), and reads for each sample were mapped back to the coassembly with bowtie2 v2.3.4.3 (86) (Text S1). The majority of the remaining analyses were performed through Anvi’o v6.2 and v7.0 (87) as previously described (9), including contig taxonomy assignment (88), gene calling (89), identification of single-copy genes (90), gene annotation (91–93), and manual binning and curation of three MAGs (Text S1). MAG quality statistics were generated using CheckM v1.1.3 (94), which also placed all three MAGs in the *Mycoplasmataceae*. More specific MAG taxonomic identification was carried out by extracting each 16S gene and matching it to ASVs from the 16S amplicon sequencing data set. To confirm the 16S gene associated with each MAG, single assembly and binning were performed as described above for select samples (162E, 166E, and 361E).

### Pangenome comparisons and phylogenomics.

The three generated mycoplasma MAGs were compared to selected *Mycoplasma* and *Ureaplasma* reference genomes (accessions available at https://github.com/jcbrealey/cestode_microbiome) and related salmonid *Mycoplasma* genomes, including “*Candidatus* Mycoplasma salmoninae salar,” generated from gut content samples obtained from eight host salmon originating from the same sample cohort analyzed in this study, “*Candidatus* Mycoplasma salmoninae mykiss” from rainbow trout (Oncorhynchus mykiss), and “*Candidatus* Mycoplasma lavaretus” from European whitefish (Coregonus lavaretus) (9). The reference FASTA reads were annotated with the Anvi’o platform, as described above, before performing comparative pangenome analyses with Anvi’o (Text S1). Pairwise average nucleotide identity (ANI) was then calculated for each genome/MAG with pyANI (95). Gene cluster and functional annotation were extracted for each genome. Additional functional annotation was performed with RAST (96, 97), taking into account *Mycoplasma* readthrough of the stop codon (98). Exploratory analysis was then performed in R v4.0.2.

To construct a phylogenomic tree, 38 additional *Mycoplasma* reference genomes were processed in Anvi’o in the same manner as above using B. pumilus as an outgroup (accessions available at https://github.com/jcbrealey/cestode_microbiome). Concatenated amino acid sequences for all HMM hits for each genome were used to generate a maximum-likelihood phylogeny using RAxML v8.2.11 (77, 78) (Text S1). The resulting tree was visualized in FigTree v1.4.3.

10.1128/mbio.00679-22 TEXT S1 Supplemental Methods. Download Text S1, PDF file, 0.2 MB. Copyright © 2022 Brealey et al. 2022 Brealey et al. https://creativecommons.org/licenses/by/4.0/ This content is distributed under the terms of the Creative Commons Attribution 4.0 International license.

### Data availability.

The raw amplicon and metagenomic sequencing data, as well as the binned metagenome assemblies of the three individual draft MAGs, have been deposited in the European Nucleotide Archive under BioProject accession no. PRJEB51496. Individual and sample metadata, ASV count data, MAG gene cluster data, additional quality control figures, and R scripts used for analysis are available on GitHub (https://github.com/jcbrealey/cestode_microbiome).
