# Supplementary figures and images for: Microbiome “Inception”: an Intestinal Cestode Shapes a Hierarchy of Microbial Communities Nested within the Host
Source: mBio. 2022 May 3;13(3):e00679-22. doi: 10.1128/mbio.00679-22 (PMC9239044; doi:10.1128/mbio.00679-22)

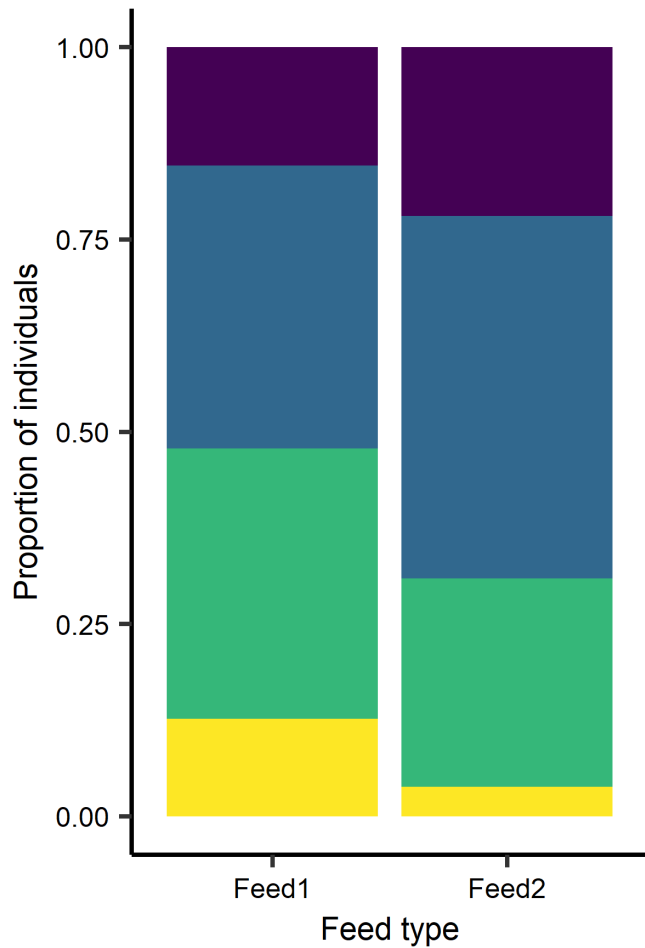

## Cestode index

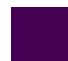

0

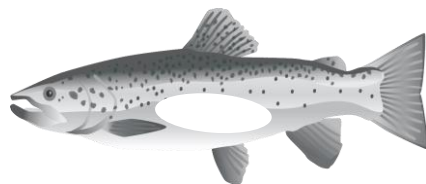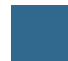

1

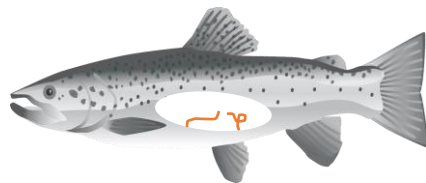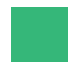

2

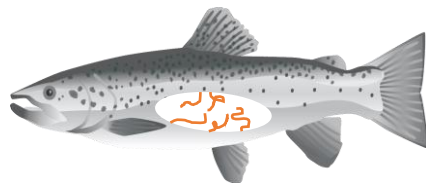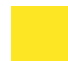

3

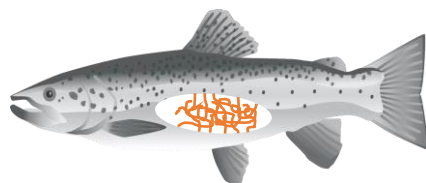

Supplement: FIG S1 [file mbio.00679-22-s0004.pdf]

**a** HoloFish cohort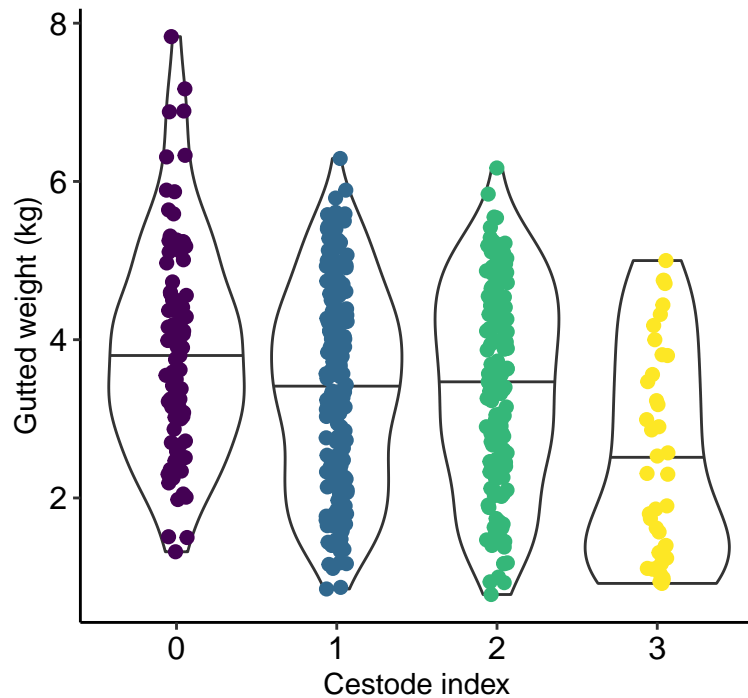**b** Cestode investigation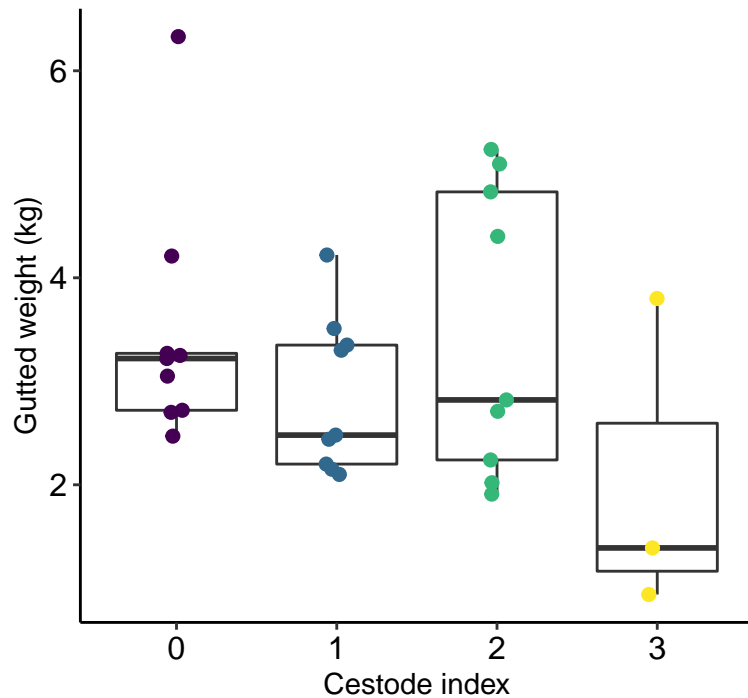

Supplement: FIG S2 [file mbio.00679-22-s0005.pdf]

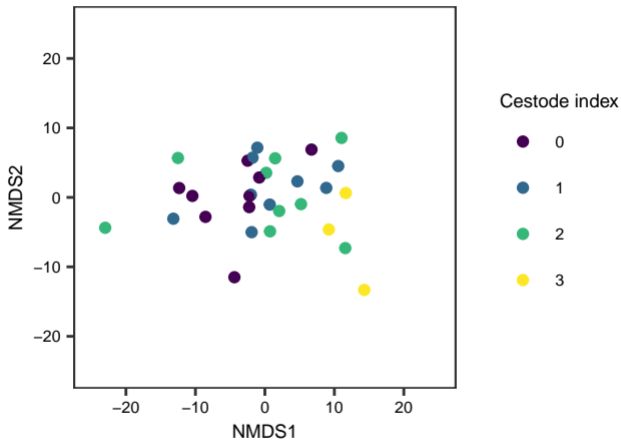

Supplement: FIG S3 [file mbio.00679-22-s0006.pdf]

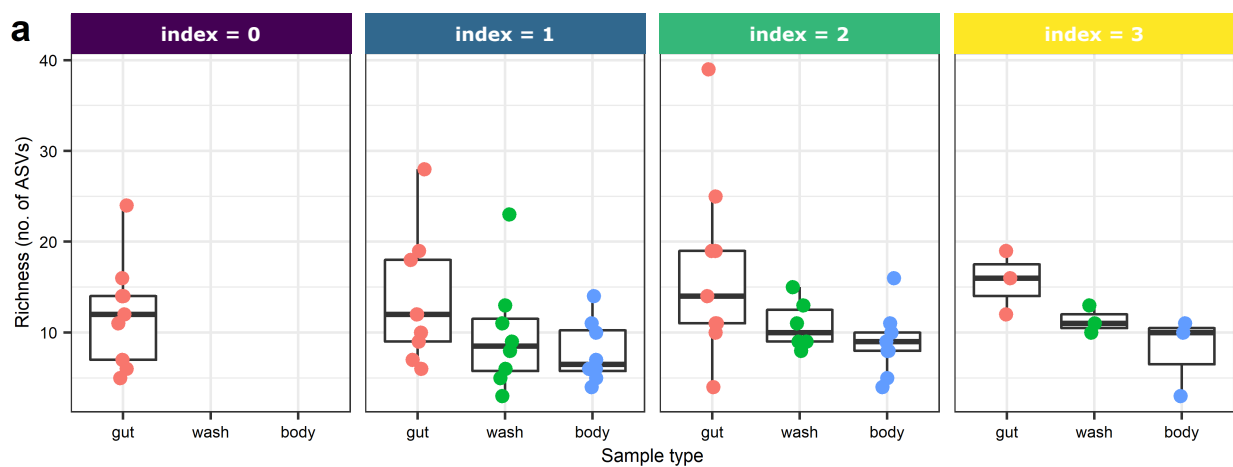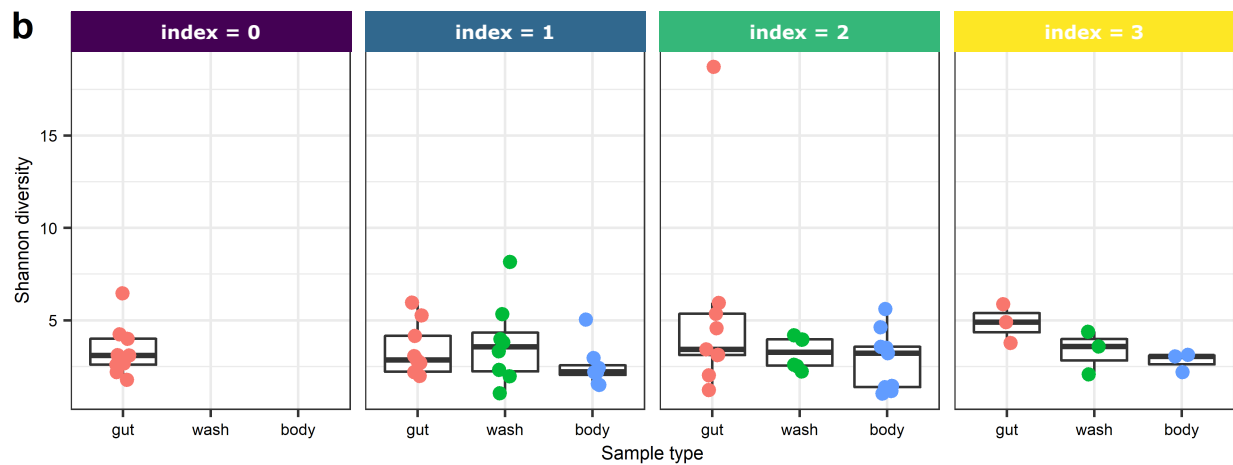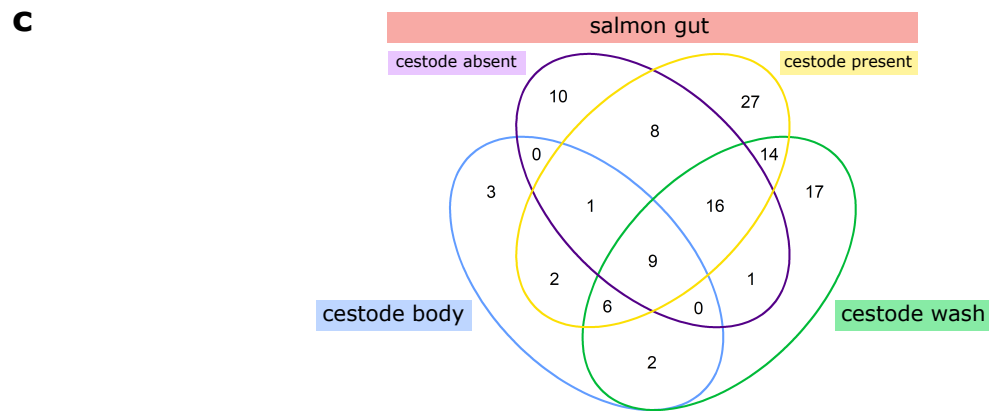

Supplement: FIG S4 [file mbio.00679-22-s0007.pdf]

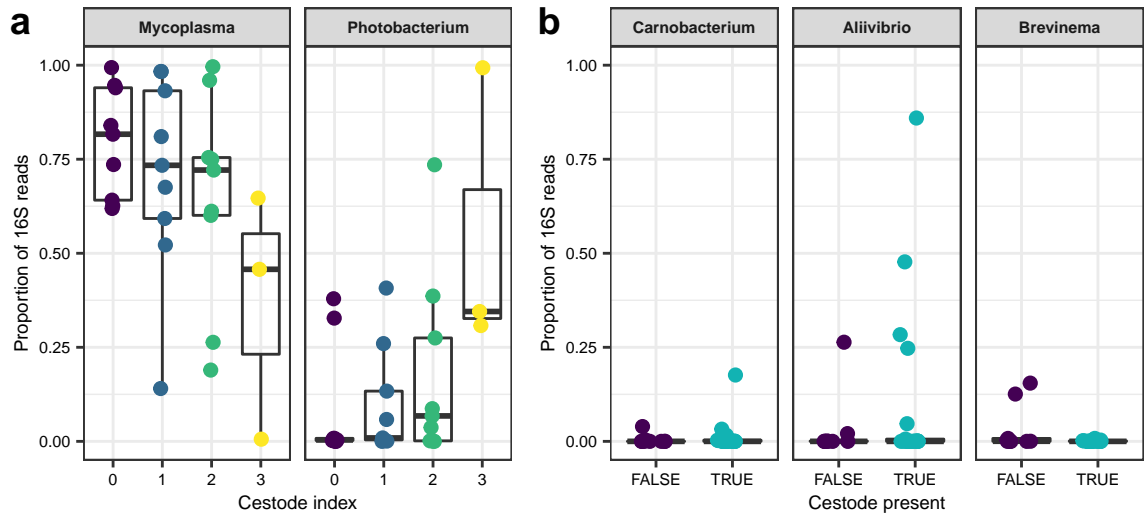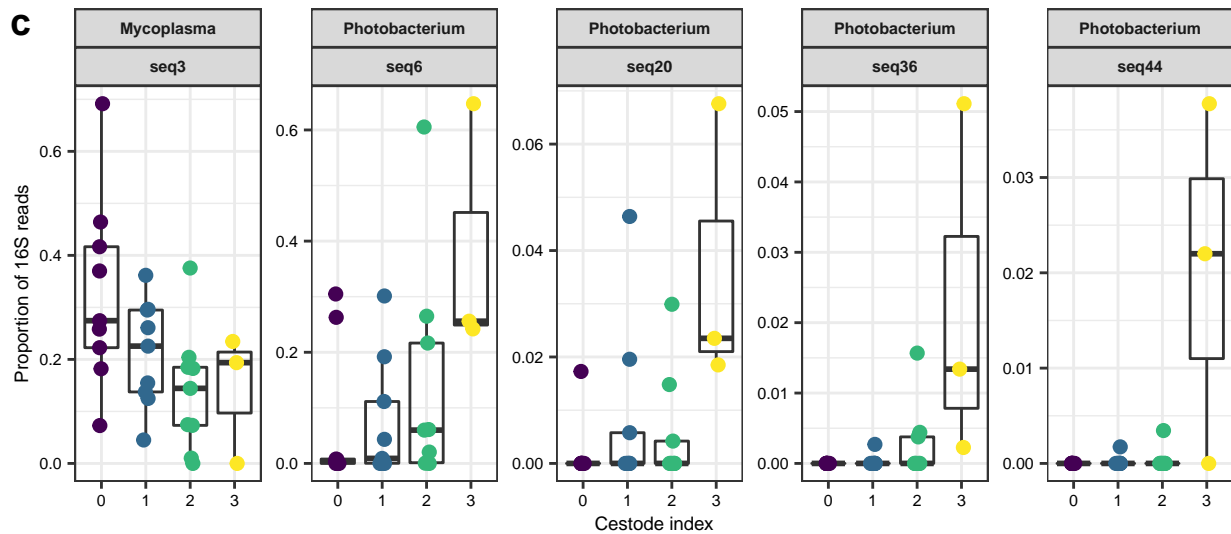

Supplement: FIG S5 [file mbio.00679-22-s0008.pdf]

**a**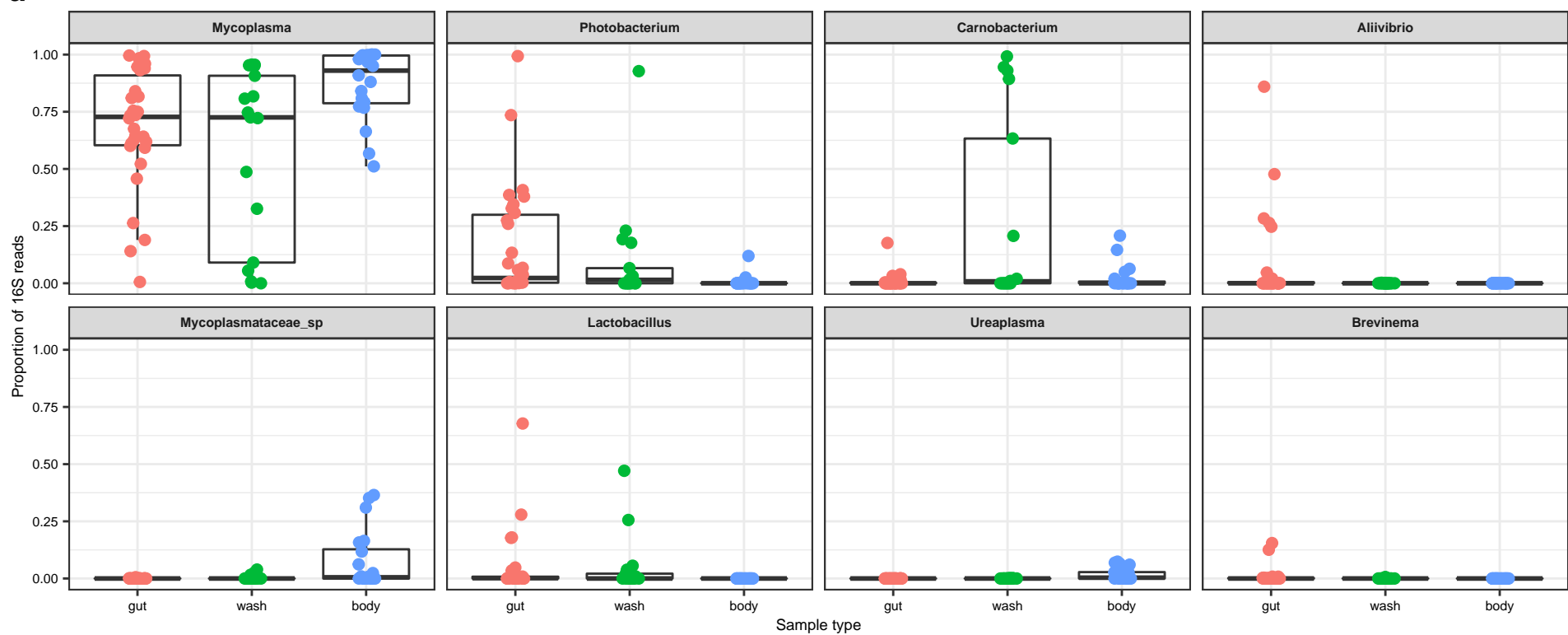**b**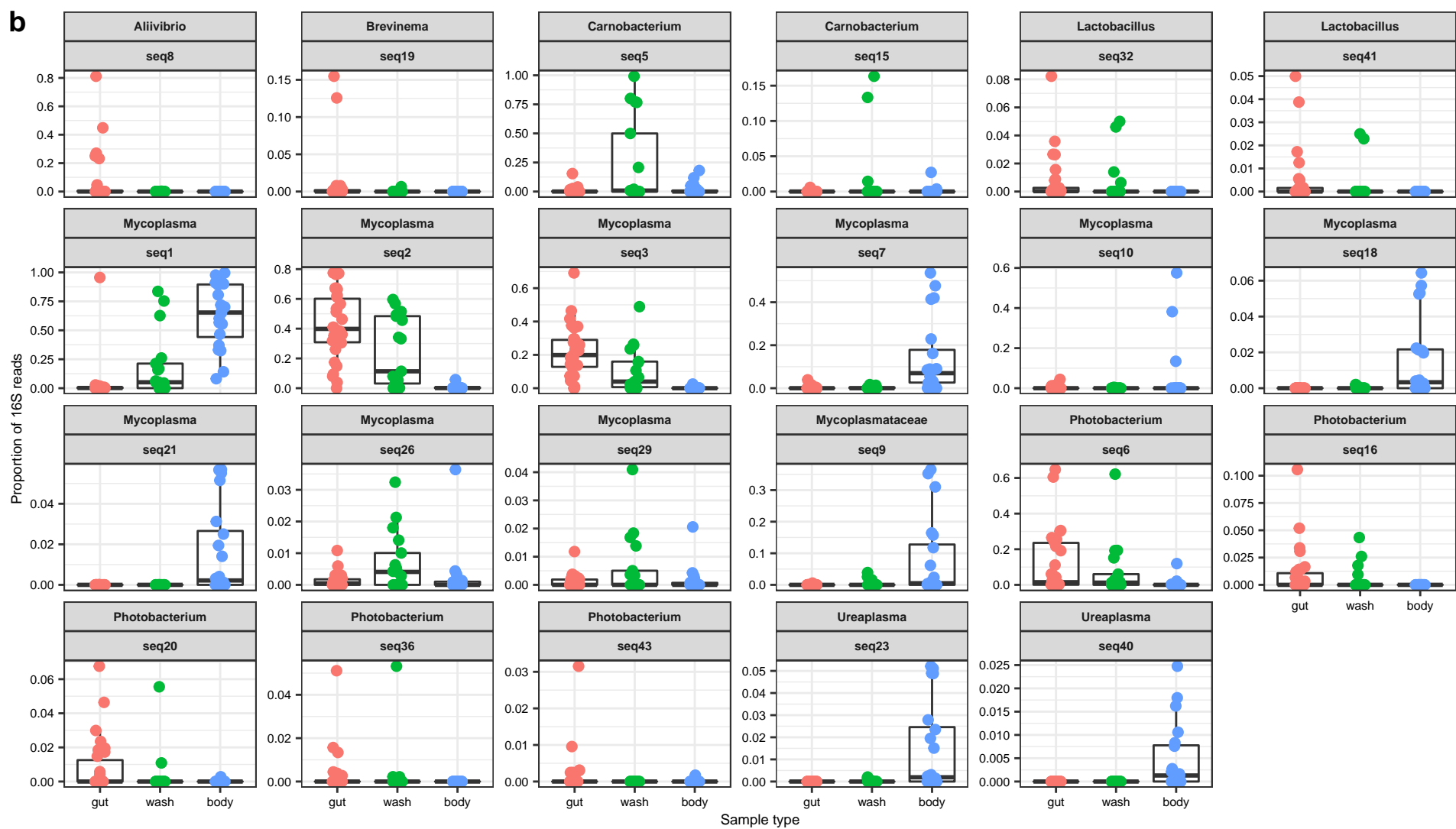

Supplement: FIG S6 [file mbio.00679-22-s0009.pdf]
