# Supplementary material for: Microbiome “Inception”: an Intestinal Cestode Shapes a Hierarchy of Microbial Communities Nested within the Host
Source: mBio. 2022 May 3;13(3):e00679-22. doi: 10.1128/mbio.00679-22 (PMC9239044; doi:10.1128/mbio.00679-22)
Supplement: TABLE S1 [file mbio.00679-22-s0002.pdf]

Table S1. Associations between ASV or genus abundance and cestode index or sample type, as produced by Maaslin2. Results with FDR < 0.25 are included and those with FDR < 0.05 are highlighted in grey.

Variable	Taxon	Level/ model type	Coeff-icient	Std error	N	N (non 0)	p-value	FDR (q-value)	Genus of ASV	Model
Cestode index	Photobacterium	linear	0.375	0.112	30	26	0.00295	0.144	NA	Genus relative abundance ~ cestode index + feed type + sex + size class + sampling date (random effect)[1]
	Mycoplasma	linear	-0.349	0.118	30	30	0.00706	0.173	NA	
Cestode index	seq44	linear	0.011	0.003	30	4	0.00047	0.105	Photobacterium	ASV relative abundance ~ cestode index + feed type + sex + size class + sampling date (random effect)[1]
	seq3	linear	-0.205	0.068	30	28	0.00663	0.247	Mycoplasma	
	seq6	linear	0.271	0.082	30	24	0.00318	0.247	Photobacterium	
	seq20	linear	0.022	0.007	30	10	0.00597	0.247	Photobacterium	
	seq36	linear	0.012	0.004	30	7	0.00574	0.247	Photobacterium	
Sample type	Photobacterium	body	-0.210	0.053	51	31	0.00036	0.006	NA	Genus relative abundance ~ sample type + fish ID (random effect) + sampling date (random effect)[2]
	Photobacterium	wash	-0.120	0.053	51	31	0.02955	0.068	NA	
	Carnobacterium	wash	0.259	0.075	51	22	0.00125	0.008	NA	
	Ureaplasma	body	0.020	0.006	51	12	0.00152	0.008	NA	

Variable	Taxon	Level/ model type	Coeff-icient	Std error	N	N (non 0)	p-value	FDR (q-value)	Genus of ASV	Model
	Mycoplasmataceae sp	body	0.070	0.024	51	16	0.00561	0.022	NA	
	Mycoplasma	body	0.235	0.094	51	50	0.01637	0.052	NA	
	Brevinema	body	-0.001	0.001	51	9	0.02352	0.063	NA	
	Brevinema	wash	-0.001	0.001	51	9	0.11969	0.186	NA	
	Aliivibrio	wash	-0.048	0.025	51	8	0.06647	0.118	NA	
	Aliivibrio	body	-0.048	0.025	51	8	0.06580	0.118	NA	
	Lactobacillus	body	-0.062	0.040	51	16	0.12818	0.186	NA	
Sample type	seq1	body	0.646	0.063	51	45	<0.0001	<0.00001	Mycoplasma	ASV relative abundance ~ sample type + fish ID (random effect) + sampling date (random effect)
	seq1	wash	0.183	0.063	51	45	0.00669	0.024	Mycoplasma	
	seq2	body	-0.426	0.063	51	42	<0.0001	0.000004	Mycoplasma	
	seq2	wash	-0.190	0.063	51	42	0.00529	0.023	Mycoplasma	
	seq3	body	-0.185	0.030	51	35	<0.0001	0.00001	Mycoplasma	
	seq3	wash	-0.079	0.030	51	35	0.01330	0.045	Mycoplasma	
	seq5	wash	0.228	0.067	51	22	0.00137	0.010	Carnobacterium	

Variable	Taxon	Level/ model type	Coeff-icient	Std error	N	N (non 0)	p-value	FDR (q-value)	Genus of ASV	Model
	seq6	body	-0.154	0.038	51	30	0.00029	0.004	Photobacterium	
	seq6	wash	-0.087	0.038	51	30	0.02931	0.083	Photobacterium	
	seq7	body	0.104	0.026	51	30	0.00034	0.004	Mycoplasma	
	seq8	wash	-0.045	0.024	51	8	0.06497	0.135	Aliivibrio	
	seq8	body	-0.046	0.024	51	8	0.06428	0.135	Aliivibrio	
	seq9	body	0.070	0.024	51	16	0.00561	0.023	Mycoplasmataceae	
	seq10	body	0.060	0.031	51	9	0.06351	0.135	Mycoplasma	
	seq15	wash	0.018	0.009	51	6	0.06062	0.135	Carnobacterium	
	seq16	body	-0.016	0.005	51	15	0.00604	0.023	Photobacterium	
	seq16	wash	-0.010	0.005	51	15	0.07151	0.143	Photobacterium	
	seq18	body	0.016	0.005	51	12	0.00159	0.010	Mycoplasma	
	seq19	body	-0.001	0.001	51	9	0.02352	0.071	Brevinema	
	seq20	body	-0.012	0.004	51	11	0.00421	0.021	Photobacterium	
	seq20	wash	-0.008	0.004	51	11	0.04474	0.121	Photobacterium	

Variable	Taxon	Level/ model type	Coeff-icient	Std error	N	N (non 0)	p-value	FDR (q-value)	Genus of ASV	Model
	seq21	body	0.016	0.005	51	8	0.00171	0.010	Mycoplasma	
	seq23	body	0.015	0.004	51	12	0.00151	0.010	Ureaplasma	
	seq26	wash	0.005	0.002	51	29	0.02380	0.071	Mycoplasma	
	seq29	wash	0.004	0.002	51	22	0.05087	0.131	Mycoplasma	
	seq32	body	-0.008	0.005	51	11	0.10336	0.183	Lactobacillus	
	seq36	body	-0.005	0.003	51	9	0.08174	0.152	Photobacterium	
	seq40	body	0.005	0.002	51	8	0.00330	0.018	Ureaplasma	
	seq41	body	-0.005	0.003	51	6	0.10497	0.183	Lactobacillus	
	seq43	wash	-0.003	0.002	51	6	0.06519	0.135	Photobacterium	
	seq43	body	-0.003	0.002	51	6	0.07481	0.144	Photobacterium	

[1] Cestode presence was not associated with any taxa (genus or ASV) at FDR < 0.2.

[2] Cestode wash and cestode body were compared to salmon gut as the base level within sampling type.
