# Supplementary material for: Microbiome “Inception”: an Intestinal Cestode Shapes a Hierarchy of Microbial Communities Nested within the Host
Source: mBio. 2022 May 3;13(3):e00679-22. doi: 10.1128/mbio.00679-22 (PMC9239044; doi:10.1128/mbio.00679-22)
Supplement: TABLE S2 [file mbio.00679-22-s0003.pdf]

Table S2. Statistics and ENA accessions of the three *Mycoplasma* MAGs generated from the cestode body shotgun metagenome.

	**CE_seq1**	**CE_seq7**	**CE_seq10**
ENA binned assembly accession	ERZ7517636	ERZ7517642	ERZ7517631
Total size (Kb)	790.88	709.84	350.56
No. of contigs	318	301	228
N50	3057	2587	1496
GC content (%)	26.18	28.41	28.26
Completeness (%)	77.69	77.98	38.03
Contamination (%) - checkM	0.77	2.08	8.05
Redundancy (%) - Anvi'o	0	5.63	4.23
Strain heterogeneity (%)	50	70	0
No. of gene clusters	741	642	349
No. of called genes (≥ 1 per cluster)	769	730	393
No. of annotated genes	455	447	277
**Mean coverage per sample**			
i162E	2.86	2.42	4.96
i166E	8.77	14.51	0.73
i169E	2.18	1.3	0.28
i343E	0.33	0.26	0.01
i347E	3.51	1.33	0.09
i348E	8.59	1.48	0.14
i350E	6.64	0.87	0.19
i361E	14.53	1.22	0.07
