# Supplementary material for: Microbiome “Inception”: an Intestinal Cestode Shapes a Hierarchy of Microbial Communities Nested within the Host
Source: mBio. 2022 May 3;13(3):e00679-22. doi: 10.1128/mbio.00679-22 (PMC9239044; doi:10.1128/mbio.00679-22)
Supplement: FIG S7 [file mbio.00679-22-s0010.pdf]

a

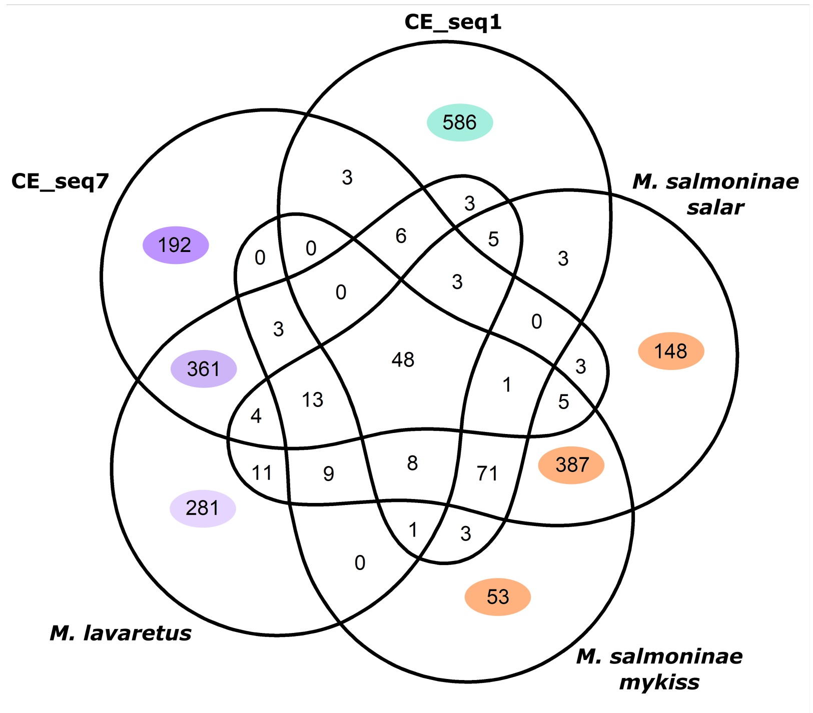

b

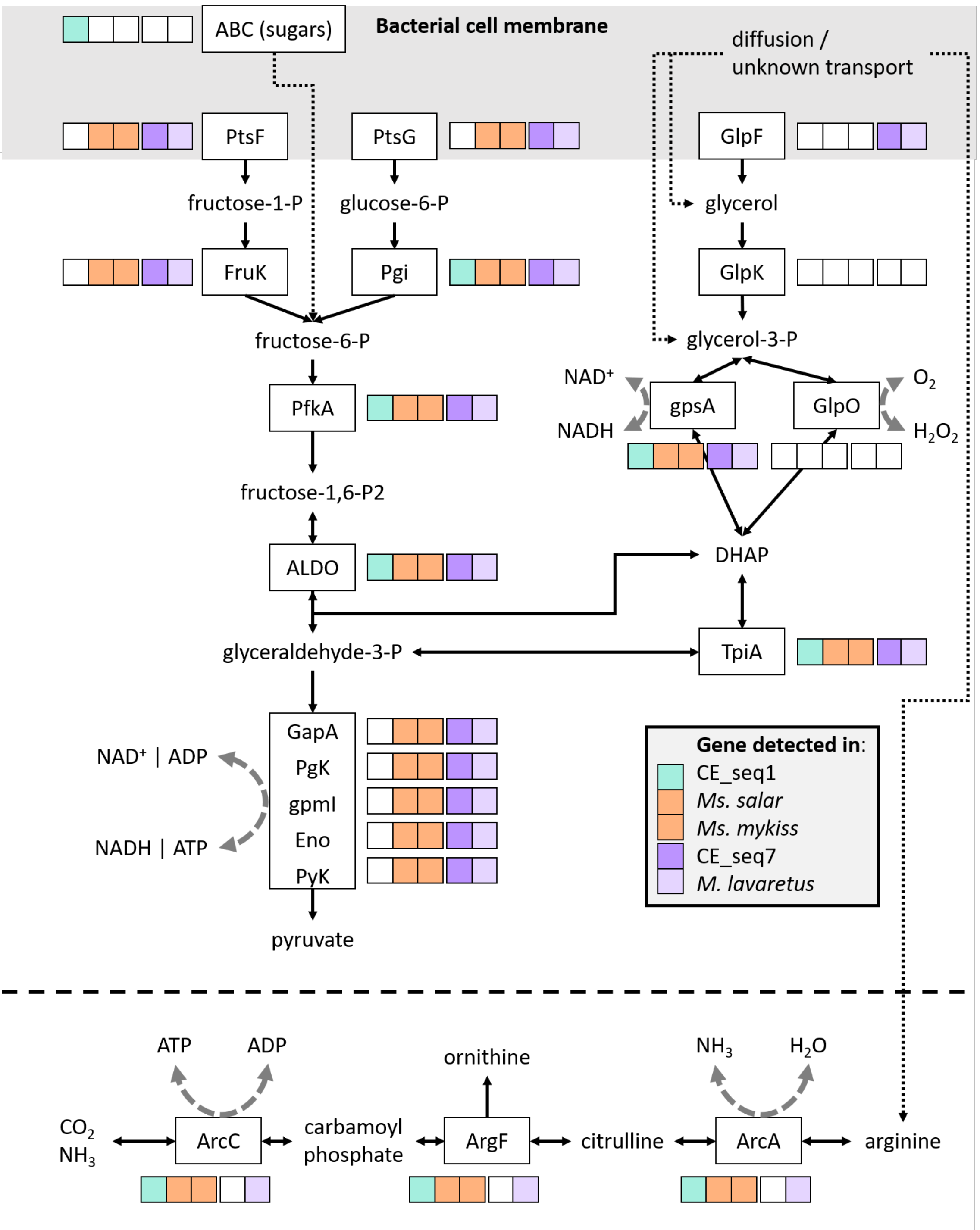

Enzyme abbreviations:

Main pathway	Abbreviation	COG20 function (accession)
sugar transport	ABC	ABC-type polysaccharide/polyol phosphate transport system (COG1134)
sugar transport	PtsF	Phosphotransferase system (PTS), fructose-specific (COG1299)
sugar transport	PtsG	Phosphotransferase system (PTS), glucose /maltose/N-acetylglucosamine-specific (COG1263)
glycolysis	FruK	1-phosphofructokinase or 6-phosphofructokinase (COG1105)
glycolysis	Pgi	Glucose-6-phosphate isomerase (COG0166)
glycolysis	PfkA	6-phosphofructokinase (COG0205)
glycolysis	ALDO	fructose/tagatose bisphosphate aldolase (COG0191)
glycolysis	GapA	glyceraldehyde-3-phosphate dehydrogenase (COG0057)
glycolysis	PgK	3-phosphoglycerate kinase (COG0126)
glycolysis	gpmI	Phosphoglycerate mutase (COG0696)
glycolysis	Eno	Enolase (COG0148)
glycolysis	PyK	Pyruvate kinase (COG0469)
glycerol	GlpF	Glycerol uptake facilitator or related aquaporin (COG0580)
glycerol	GlpK	Glycerol kinase (COG0554)
glycerol	gpsA	Glycerol-3-phosphate dehydrogenase (COG0240)
glycerol	GlpO	alpha-glycerophosphate oxidase (K00105)
glycolysis	TpiA	Triosephosphate isomerase (COG0149)
arginine deiminase	ArcC	Carbamate kinase (COG0549)
arginine deiminase	ArgF	Ornithine carbamoyltransferase (COG0078)
arginine deiminase	ArcA	Arginine deiminase (COG2235)
