# Supplementary material for: Microbiome “Inception”: an Intestinal Cestode Shapes a Hierarchy of Microbial Communities Nested within the Host
Source: mBio. 2022 May 3;13(3):e00679-22. doi: 10.1128/mbio.00679-22 (PMC9239044; doi:10.1128/mbio.00679-22)
Supplement: TEXT S1 [file mbio.00679-22-s0001.pdf]

Supplemental Methods

Sampling of salmon

Salmon were killed immediately upon catching with a solid hit to the neck region, resulting in instant death, before samples were taken, in accordance with Norwegian regulations (FOR-2015-06-18-761, Appendix C, cf. nr 16, The Norwegian Ministry of Agriculture and Food). These salmon were part of a normal commercial production cycle at one of Lerøy's licensed aquaculture facilities. While no ethical approval is required from the Norwegian Animal Research Authority for this type of animal sampling (FOR-2015-06-18-761, nr 6), at all times fish were handled following standard operating procedures under supervision by experienced and trained staff at Lerøy, in accordance with normal and legal procedures.

Several salmon traits were measured at time of sampling, including gutted weight (kg) and sex. Sex was inferred based on visual inspection by trained staff. However, the majority of individuals were not ripe, increasing uncertainty of scored sex; thus, while we include sex in statistical models, these results are interpreted with caution.

16S library builds

To amplify the V3-V4 region of the 16S ribosomal RNA gene, we used bacteria-specific custom primers, modified from the standard 341 F and 806 R primers (1), for a two-step PCR-based approach with Nextera dual indexes (Illumina):

NT_341 F (5'-TCGTCGGCAGCGTCAGATGTGTATAAGAGACAGCCTAYGGGRBGCASCAG-3')

NT_806 R (5'- GTCTCGTGGGCTCGGAGATGTGTATAAGAGACAGGGACTACNNGGGTATCTAAT-3')

Libraries were prepared using two-step PCR in duplicate to control for putative bias resulting from random PCR noise. In the first PCR reaction, we used a total volume of 25 µl per reaction, consisting of 12.5 µl of AccuStart II PCR ToughMix (Quantabio), 0.75 µl of each primer, 6 µl of molecular biology-grade H_2O (VWR), and 5 µl of DNA template. Cycling conditions for the amplification of 16S V3-V4 region consisted of an initial denaturation step for 10 min at 95°C, then 35 cycles of 15 s at 95°C, 20 s at 53°C, and 40 s at 72°C, followed

by a final extension step of 7 min at 72°C. The resulting PCR products were visualised with 2% agarose gel electrophoresis to check for successful amplification of the targeted amplicon. Failed samples were re-amplified from the extracts using 40 cycles during PCR. The resulting PCR products were purified using SPRI bead purification (2) with a beads-to-sample ratio of 1.12X, two washing steps in 200 µl of 80% EtOH and final elution in 25 µl molecular biology-grade H_2O. Nextera dual indexes (index sequences available on GitHub) were incorporated in the second 25 µl PCR reaction, consisting of 12.5 µl of AccuStart II PCR ToughMix, a unique combination of 2.5 µl of Nextera index 1 i7 (10 nmol ml−1; Illumina) and 1 µl of Nextera index 2 i5 (10 nmol ml−1; Illumina), 4 µl of molecular biology-grade H_2O, and 5 µl of the purified PCR product from the previous step. The cycling conditions consisted of an initial denaturation step of 1 min at 95°C, followed by eight cycles of 10 s at 95°C, 20 s at 55°C, 30 s at 72°C, and a final extension step of 10 min at 72°C. The resulting PCR products were purified using SPRI beads as above. After purification, the DNA concentration of each library was measured with a Qubit 2.0 fluorometer (Thermo-Fisher Scientific) following the manufacturer's guidelines. We subsequently pooled the constructed libraries in equimolar ratios based on their DNA concentrations. The positive and negative controls were included through the entire library preparation process and incorporated in the pools for downstream quality control and contaminant filtering.

16S amplicon sequence variant processing with DADA2

Quality filtering and trimming was performed with *filterAndTrim* (parameters: *truncLen=0, maxEE=c(3,3), truncQ=2, maxN=0, trimLeft=c(17,20), minLen=200, rm.phix=TRUE*). Samples were dereplicated using *derepFastq* with default parameters. To infer amplicon sequence variants (ASVs), forward and reverse error rates were learned with the function *learnErrors* using a subset of 10^8 bases to increase speed (*nbases=1e8*). Sequence variants for forward and reverse reads were then inferred using the *dada* function with default parameters. Paired-end reads were merged using *mergePairs* (parameters: *minOverlap=12, maxMismatch=2*). An ASV abundance table was then constructed with *makeSequenceTable* using default parameters. *De novo* chimeras were filtered from the ASV table using *removeBimeraDenovo* (*method="consensus"*). Taxonomy was then assigned at the genus level using the function *assignTaxonomy* with default parameters and a custom database (accessions available on GitHub) based on the SILVA

nonredundant SSU v138 training set provided by DADA2 (https://zenodo.org/record/3731176/files/silva_nr_v138_train_set.fa.gz, accessed 13-08-2020). Species assignments at 100% sequence identity were performed where possible with the function *addSpecies*.

Metagenomic data processing and MAG generation

For the shotgun metagenomics data, adapters were removed with AdapterRemoval v2.3.1 (3) and reads < 50 bp filtered out. Identical PCR duplicates were removed from forward and reverse reads using seqkit v0.8.0 *rmdup* (4) and bbmap v38.70 *repair* (https://sourceforge.net/projects/bbmap/). Reads mapping to the sequencing control phiX174 were removed by aligning to the reference genome NC_001422.1 with bwa v0.7.17 mem (5, 6). Unmapped reads were retained with samtools v1.9 *view* (7) and fastq reads extracted from the BAM files with the bedtools v2.29.0 function *bamtofastq* (8). Reads mapping to the salmon reference genome GCF_000233375.1 (9), a consensus cestode reference consisting of 24 publicly available Eucestoda genomes (accessions available on GitHub) and the human reference genome (GCF_000001405.39_GRCh38.p13) were removed in the same manner.

A coassembly of reads from all samples was then performed with MEGAHIT v1.1.1 (10), using a minimum contig length of 1000 bp and the meta-sensitive preset (a set of parameters aimed at providing more sensitive results). Reads for each sample were mapped back to the coassembly with bowtie2 v2.3.4.3 (11). The majority of the remaining analyses were performed through Anvi'o v6.2 & v7.0 (12), as previously described (13). Briefly, open reading frames were identified with Prodigal (14) and single copy genes were identified using HMMs (15) against the Anvi'o default database. Annotation was performed using the NCBI Clusters of Orthologous Genes (COGs), Pfam and KEGG Orthology (KO) databases (16–18). Taxonomy assignment of contigs was performed with Kraken2 v2.0.9-beta (19) using the Kraken2 nt database (built 14-08-2020). Samples were profiled with Anvio's *anvi-profile* using a minimum contig length of 1000 bp and merged for comparison with *anvi-merge*, including hierarchical clustering of contigs. Contig clustering was then visualised with *anvi-interactive* and manual binning and curation of three MAGs was performed based on differential coverage across samples, GC% and Anvio's contig clustering. MAG quality statistics were

generated using CheckM v1.1.3 (20) following the lineage-specific workflow, which also placed all three MAGs in the *Mycoplasmataceae*. More specific MAG taxonomic identification was carried out by extracting each 16S gene and matching it to ASVs generated during the 16S amplicon sequencing. To confirm the 16S gene associated with each MAG, single assembly and binning was performed as above for select samples (162E, 166E and 361E).

Pangenome comparisons

We then compared the three generated mycoplasma MAGs to selected *Mycoplasma* and *Ureaplasma* reference genomes (accessions available on GitHub) and related salmonid *Mycoplasma* genomes: 'Candidatus' *Mycoplasma salmoninae salar*, generated from gut content samples obtained from eight host salmon originating from the same sample cohort analysed in this study, 'Candidatus' *Mycoplasma salmoninae mykiss* from rainbow trout (*Oncorhynchus mykiss*) and 'Candidatus' *Mycoplasma lavaretus* from European whitefish (*Coregonus lavaretus*) (13). Anvi'o contig databases were generated and annotated from the reference fasta reads as above. All contig databases were then used to generate an Anvi'o pangenome database using *anvi-pan-genome* (parameters: *--use-ncbi-blast --mcl-inflation 2 --enforce-hierarchical-clustering*). Pairwise Average Nucleotide Identity (ANI) was then calculated for each genome/MAG using *anvi-compute-genome-similarity* with pyANI (21). Gene cluster and functional annotation was extracted for each genome and KEGG pathway completeness was calculated with *anvi-estimate-metabolism*. Additional functional annotation was performed with RAST (22, 23), taking into account *Mycoplasma* readthrough of the stop codon (24). Exploratory analysis was then performed in R v4.0.2.

To construct a phylogenomic tree, 38 additional *Mycoplasma* reference genomes were processed in Anvi'o in the same manner as above using *B. pumilus* as an outgroup (accessions available on GitHub). Concatenated amino acid sequences for all HMM hits for each genome were extracted using *anvi-get-sequences-for-hmm-hits* and Anvio's 'Bacteria_71' database. A maximum likelihood phylogeny was then generated using RAxML v8.2.11 (25, 26) *raxmlHPC-PTHREADS-SSE3* (for multi-threading) with 100 rapid bootstrap searches using

automatic determination of the most appropriate protein model (parameters: *-f a -m PROTGAMMAAUTO -# 100*). The resulting tree was visualised in FigTree v1.4.3.

Supplementary references

1. Yu Y, Lee C, Kim J, Hwang S. 2005. Group-specific primer and probe sets to detect methanogenic communities using quantitative real-time polymerase chain reaction. Biotechnol Bioeng 89:670–679.

2. DeAngelis MM, Wang DG, Hawkins TL. 1995. Solid-phase reversible immobilization for the isolation of PCR products. Nucleic Acids Res 23:4742–4743.

3. Schubert M, Lindgreen S, Orlando L. 2016. AdapterRemoval v2: Rapid adapter trimming, identification, and read merging. BMC Res Notes 9:88.

4. Shen W, Le S, Li Y, Hu F. 2016. SeqKit: A Cross-Platform and Ultrafast Toolkit for FASTA/Q File Manipulation. PLoS One 11:e0163962.

5. Li H. 2013. Aligning sequence reads, clone sequences and assembly contigs with BWA-MEM. arXiv preprint arXiv:1303.3997.

6. Li H, Durbin R. 2009. Fast and accurate short read alignment with Burrows-Wheeler transform. Bioinformatics.

7. Li H, Handsaker B, Wysoker A, Fennell T, Ruan J, Homer N, Marth G, Abecasis G, Durbin R, 1000 Genome Project Data Processing Subgroup. 2009. The Sequence Alignment/Map format and SAMtools. Bioinformatics 25:2078–2079.

8. Quinlan AR, Hall IM. 2010. BEDTools: A flexible suite of utilities for comparing genomic features. Bioinformatics 26:841–842.

9. Lien S, Koop BF, Sandve SR, Miller JR, Kent MP, Nome T, Hvidsten TR, Leong JS, Minkley DR, Zimin A, Grammes F, Grove H, Gjuvsland A, Walenz B, Hermansen RA, Von Schalburg K, Rondeau EB, Di Genova A, Samy JKA, Olav Vik J, Vigeland MD, Caler L, Grimholt U, Jentoft S, Inge Våge D, De Jong P, Moen T, Baranski M, Palti Y, Smith DR, Yorke JA, Nederbragt AJ, Tooming-Klunderud A, Jakobsen KS, Jiang X, Fan D, Hu Y, Liberles DA, Vidal R, Iturra P, Jones SJM, Jonassen I, Maass A, Omholt SW, Davidson WS. 2016. The Atlantic salmon genome provides insights into rediploidization. Nature 533:200–205.

10. Li D, Liu C-M, Luo R, Sadakane K, Lam T-W. 2015. MEGAHIT: an ultra-fast single-node solution for large and complex metagenomics assembly via succinct de Bruijn graph. Bioinformatics 31:1674–1676.

11. Langmead B, Salzberg SL. 2012. Fast gapped-read alignment with Bowtie 2. Nat Methods 9:357–359.

12. Eren AM, Esen OC, Quince C, Vineis JH, Morrison HG, Sogin ML, Delmont TO. 2015. Anvi'o: An advanced analysis and visualization platformfor 'omics data. PeerJ 3:e1319.

13. Rasmussen JA, Villumsen KR, Duchene D, Puetz LC, Delmont TO, Sveier H, Præbel K, Jørgensen L von G, Martin MD, Gilbert MTP, Bojesen AM, Kristiansen K, Limborg MT. 2021. Genome-resolved metagenomics suggests a mutualistic relationship between Mycoplasma and salmonid hosts. Communications Biology 4:579.

14. Hyatt D, Chen GL, LoCascio PF, Land ML, Larimer FW, Hauser LJ. 2010. Prodigal: Prokaryotic gene recognition and translation initiation site identification. BMC Bioinformatics 11:119.

15. Lee MD. 2019. GToTree: a user-friendly workflow for phylogenomics. Bioinformatics 35:4162–4164.

16. Galperin MY, Wolf YI, Makarova KS, Vera Alvarez R, Landsman D, Koonin EV. 2021. COG database update: focus on microbial diversity, model organisms, and widespread pathogens. Nucleic Acids Res 49:D274–D281.

17. Mistry J, Chuguransky S, Williams L, Qureshi M, Salazar GA, Sonnhammer ELL, Tosatto SCE, Paladin L, Raj S, Richardson LJ, Finn RD, Bateman A. 2021. Pfam: The protein families database in 2021. Nucleic Acids Res 49:D412–D419.

18. Kanehisa M, Furumichi M, Sato Y, Ishiguro-Watanabe M, Tanabe M. 2021. KEGG: integrating viruses and cellular organisms. Nucleic Acids Res 49:D545–D551.

19. Wood DE, Lu J, Langmead B. 2019. Improved metagenomic analysis with Kraken 2. Genome Biol 20:257.

20. Parks DH, Imelfort M, Skennerton CT, Hugenholtz P, Tyson GW. 2015. CheckM: assessing the quality of microbial genomes recovered from isolates, single cells, and metagenomes. Genome Res 25:1043–1055.

21. Pritchard L, Glover RH, Humphris S, Elphinstone JG, Toth IK. 2015. Genomics and taxonomy in diagnostics for food security: soft-rotting enterobacterial plant pathogens. Anal Methods 8:12–24.

22. Aziz RK, Bartels D, Best AA, DeJongh M, Disz T, Edwards RA, Formsma K, Gerdes S, Glass EM, Kubal M, Meyer F, Olsen GJ, Olson R, Osterman AL, Overbeek RA, McNeil LK, Paarmann D, Paczian T, Parrello B, Pusch GD, Reich C, Stevens R, Vassieva O, Vonstein V, Wilke A, Zagnitko O. 2008. The RAST Server: rapid annotations using subsystems technology. BMC Genomics 9:75.

23. Overbeek R, Olson R, Pusch GD, Olsen GJ, Davis JJ, Disz T, Edwards RA, Gerdes S, Parrello B, Shukla M, Vonstein V, Wattam AR, Xia F, Stevens R. 2014. The SEED and the Rapid Annotation of microbial genomes using Subsystems Technology (RAST). Nucleic Acids Res 42.

24. Inamine JM, Ho KC, Loechel S, Hu PC. 1990. Evidence that UGA is read as a tryptophan codon rather than as a stop codon by Mycoplasma pneumoniae, Mycoplasma genitalium, and Mycoplasma gallisepticum. J Bacteriol 172:504–506.

25. Stamatakis A. 2014. RAxML version 8: A tool for phylogenetic analysis and post-analysis of large phylogenies. Bioinformatics 30:1312–1313.

26. Stamatakis A, Hoover P, Rougemont J. 2008. A rapid bootstrap algorithm for the RAxML Web servers. Syst Biol 57:758–771.
